# Identification of Natural Compounds against Neurodegenerative Diseases Using In Silico Techniques

**DOI:** 10.3390/molecules23081847

**Published:** 2018-07-25

**Authors:** Larisa Ivanova, Mati Karelson, Dimitar A. Dobchev

**Affiliations:** Institute of Chemistry, University of Tartu, Ravila 14a, 50411 Tartu, Estonia; larisa.ivanova@ut.ee (L.I.); mati.karelson@ut.ee (M.K.)

**Keywords:** natural compounds, artificial neural networks, molecular docking, TrkA, NMDA, LRRK2, molecular dynamics, CADD

## Abstract

The aim of this study was to identify new potentially active compounds for three protein targets, tropomyosin receptor kinase A (TrkA), *N*-methyl-d-aspartate (NMDA) receptor, and leucine-rich repeat kinase 2 (LRRK2), that are related to various neurodegenerative diseases such as Alzheimer’s, Parkinson’s, and neuropathic pain. We used a combination of machine learning methods including artificial neural networks and advanced multilinear techniques to develop quantitative structure–activity relationship (QSAR) models for all target proteins. The models were applied to screen more than 13,000 natural compounds from a public database to identify active molecules. The best candidate compounds were further confirmed by docking analysis and molecular dynamics simulations using the crystal structures of the proteins. Several compounds with novel scaffolds were predicted that could be used as the basis for development of novel drug inhibitors related to each target.

## 1. Introduction

A natural product (NP) is generally defined as a chemical compound or substance that is produced by living organisms. NPs can be classified by many criteria and characteristics, such as source, biological function, biosynthetic pathway, physical and chemical properties, etc. Nowadays, NPs find a broad spectrum of applications related to human life, including an important role in medicine. Notably, the use of natural products as medicines has been described throughout human history in terms of substances related to herbal medicines, potions, oils, remedies, and foods. Many of these substances have been discovered by trial and error, and through the years they have become standard products in human lives [1,2].

In the past few decades, natural products have been an important source of potential drug hits and leads [3,4]. However, development efforts in NP drug discovery have demonstrated a certain downturn in recent years [3]. Despite this decline, the vast chemical space of natural products continues to provide abundant structural diversity for discovering novel lead compounds with low molecular weight. Less than 10% of the world’s biodiversity has been explored to find potential biologically active compounds [5,6]. Therefore, numerous lead NPs that may be used against a broad spectrum of diseases await discovery.

Many drugs related to diseases such as malaria, tuberculosis, cancer, human immunodeficiency virus (HIV), inflammatory diseases, cardiac diseases, diabetes, etc. have been derived from NPs [7,8]. One area where natural compounds have also been found to have great research potential and application is neurodegenerative diseases such as Alzheimer’s disease (AD), Parkinson’s disease (PD), schizophrenia, dementia, and neuropathic disorders [9,10,11,12,13]. Recent research in this area has indicated that *N*-methyl-d-aspartate (NMDA) receptor, leucine-rich repeat kinase 2 (LRRK2), and tropomyosin receptor kinase A (TrkA) have great potential as drug development targets for the above diseases. It has been shown that mutations in LRRK2 are the most common cause of PD [14]. Expression analysis of alpha-synuclein and LRRK2 mRNA levels indicated a significant increase in the temporal cortex of variants of AD patients’ brains as compared with control patients [15]. It is known that AD is characterized by synaptic loss, deposition of Aβ plaques, neurofibrillary tangles, and hyperphosphorylated tau. These changes are associated with NMDA receptor activation and oxidative stress, which ultimately result in AD pathology [16,17]. Moreover, NMDA receptors present in the striatum are crucial for dopamine–glutamate interactions. As such, NMDAs have been frequently used for pharmacological treatment of PD [18] The importance of tyrosine kinase receptors is well known for interactions in neuronal survival, together with nerve growth factors (NGFs). It is now known that NGF is also associated with Alzheimer’s disease and intractable pain, hence it, along with its high-affinity receptor tropomyosin receptor kinase A, is considered to be a new target for therapies being developed to treat these diseases. Anti-NGF antibody and TrkA inhibitors are known to suppress NGF/TrkA signaling. Moreover, it should be mentioned that local anesthetics also possess TrkA inhibitory effects. Therefore, the TrkA receptor plays an important role as a target for treating neurodegenerative diseases [19,20,21].

In the last two decades, statistics have confirmed that natural products can be regarded as an important source for the successful development of new drugs. However, the interest in finding new bioactive NP candidates for neurodegenerative diseases among the main pharmaceutical companies has generally declined. The main reason for this decline is that research in this field is time-consuming, expensive, highly complex, and in many cases ineffective. Therefore, a more effective and rational approach is needed. In this regard, in silico methods and related computer-aided drug design (CADD) methods have been proven throughout the years to be fast, inexpensive, and effective in tackling the above problems [22]. In particular, quantitative structure–activity relationship (QSAR) combined with molecular docking and molecular dynamics could be a powerful tool for development of new lead drug candidates. Usually the standard QSAR models are based on multilinear regression (MLR) methods. However, nonlinear modeling based on machine learning approaches such as artificial neural networks (ANNs) [23,24,25] has become important for QSAR. It has been applied in numerous areas of chemistry and pharmacy [26,27,28]. The mathematical adaptability of ANNs makes them a powerful tool for pattern classification and function/regression approximation [29]. The main advantage of ANNs is their inherent ability to incorporate nonlinear dependencies between dependent and independent variables without using an explicit mathematical function. In contrast with the standard two-dimensional (2D) QSAR approach, molecular docking and molecular dynamics make use of the three-dimensional (3D) structure of the target protein in order to investigate the specific structural characteristics and interactions between the ligand and the protein. The main purpose of molecular docking is to reveal the predominant binding modes of a ligand with the known 3D structure of a receptor. Therefore, the method can identify correct poses of ligands in the binding pocket of a protein and predict the affinity between the ligand and the protein [30,31]. On the other hand, the molecular dynamics approach is a computerized simulation that investigates the actual/physical movements of the atoms and molecules in certain media. Consequently, this method describes patterns, strengths, and properties of protein behavior, drug-receptor interactions, the solvation of molecules, the conformational changes that a protein or molecule may undergo under various conditions, and other events that require the systematic evaluation of molecular properties in dynamic molecular systems [32,33,34].

A non-exhaustive search in the recent scientific literature shows that there are not many works that deal with the exact problem as the present study, i.e., in silico modeling of natural compounds for neurodegenerative diseases. One such work is by Ambure et al., who successfully developed linear discriminant analysis (LDA) related to Alzheimer’s disease [35]. The classification models were used for virtual screening of large numbers of NPs. The best candidates predicted by the LDA classification models were submitted to molecular docking and molecular dynamics. Also, the paper by Corrêa and Fernandes studied the QSAR works related to discovery of potential drugs related to histamine H3R receptor [36]. The H3R is an important target involved in several CNS disorders, such as narcolepsy, attention deficit hyperactivity disorder and schizophrenia. The authors concluded that the QSAR methods are valuable to design better H3R antagonists/inverse agonists. However, pharmacokinetics should also be considered in the models to ensure good CNS penetration. In addition, the reader is also pointed to the review article by Nikolic et al. and the references therein describing works related to discovery of CNS drugs based chemoinformatics, 3D-QSAR and virtual screening techniques [37].

The aim of this study was twofold: (i) to develop potential natural inhibitor hits for multiple protein targets (NMDA, LRRK2, TrkA) related to neurodegenerative diseases (AD, PD), and (ii) to demonstrate the usefulness of a hybrid approach comprising QSAR (machine learning: ANN, MLR, virtual screening) and molecular modeling (molecular docking and molecular dynamics) as applied to the natural compound space. Therefore, we first developed three ANN and three MLR models for the related protein targets. These models were used for further virtual screening of databases of natural compounds to find possible active inhibitors. Then the best candidates were submitted for molecular docking analysis regarding the respective proteins. The best refined candidates from the docking were used in molecular dynamics for further investigation of the molecular interactions. Finally, we identified several possible candidates that passed successfully through the above computational flow.

## 2. Results and Discussion

### 2.1. Nonlinear QSAR 

Several back-propagation neural network models with different architectures were investigated for each target set. In all our models, the single output was assigned to logIC_50_ and the asociated experimental values were normalized within the [−1, 1] range. The generalized delta rule was used for updating the weights of the ANN where two adjustable parameters had to be defined prior to the training procedure, namely momentum (alpha) and learning rate (eta). The best ANN models developed for the three sets related to the different targets are shown in Table 1, with their statistical parameters and characteristics. The notations in the headings of Table 1 are as follows: Target, network model name for the respective receptor; alpha, momentum; eta, learning rate; N_tr_, number of training data; N_val_, number of validation data; epoch, final training epoch; R^2^_tr_, coefficient of determination for the training set; R^2^_val_, coefficient of determination for the validation set; RMS_tr_, root mean squared error for the training set; RMS_val_, root mean squared error for the validation set; Architecture, connected network layers and number of neurons within each layer; Input Descriptors, descriptor names in the input layer (independent variables).

**Figure 1:**
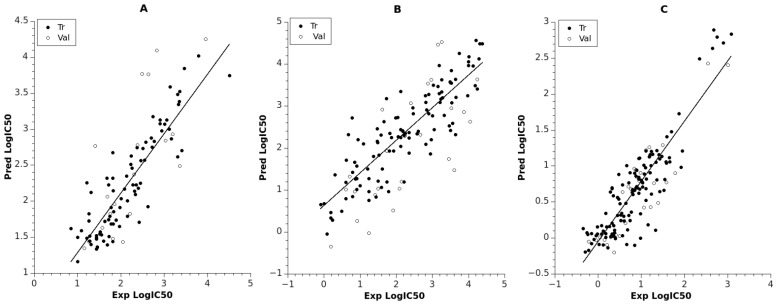
Predicted vs. experimental logIC_50_ for training and validation sets: (**A**) ANN model for LRRK2; (**B**) ANN model for NMDA; (**C**) ANN model for TrkA. Trend lines used for training data.

The model for LRRK2 had two hidden layers, with five neurons in the first and three neurons in the second hidden layer. The corresponding RMS_tr_ = 0.195 (R^2^_tr_ = 0.788) for the training set indicated satisfactory prediction, while the large statistical variation of the logIC_50_ [0.845, 4.505] for the set led to RMS_val_ = 0.381. The linear fit between the predicted and experimental logIC_50_ is shown in Figure 1A (see also Supplementary Table S1). For this model, the most important descriptors (ANN inputs) are related to the reactivity of the compounds (maximal nucleophilic reactivity index (AM1) for C atoms, average electrophilic reactivity index (AM1) for C atoms, maximal electrophilic reactivity index (AM1) for N atoms) and are derived from molecular orbital theory. Also, the descriptors highest coulombic interaction (AM1) and HPSA (Hydrogen polar surface area) polar (AM1), part of the solvent accessible surface area, play an important role in the electrostatic attraction/repulsion within the intermolecular distances. Moreover, it is noticable that compounds with larger highest coulombic interaction (AM1) values lead to more active compounds, which is reflected by the negative correlation with logIC_50_ (R = −0.564) for the whole dataset.

The model for NMDA included larger datasets (N_tr_ = 107, N_val_ = 26) than the model for LRRK2 (N_tr_ = 81, N_val_ = 20). The prediction quality for the training set was RMS_tr_ = 0.273 (R^2^_tr_ = 0.752), while for the validation set it was RMS_val_ = 0.444 (R^2^_val_ = 0.519), as indicated in Table 1. A linear plot of the experimental and calculated predictions is shown in Figure 1B, and their values are collected in Supplementary Table S2. The model for NMDA had the same number of layers as the model for LRRK2 but included more neurons in the second hidden layer in order to encounter the huge data variability in logIC_50_ for the whole set (almost 5 log units). The molecular features selected for the inputs of the NMDA network were attributed to (i) compactness/branchness of the compound structure (Kier and Hall index (order 2)); (ii) charged molecular areas (charged (Zefirov) surface area of O atoms, square root of charged (Zefirov) surface area of N atoms); (iii) reactivity of the compounds based on O atoms (minimal nucleophilic reactivity index (AM1) for O atoms, highest e-e repulsion (AM1) for C–O bonds). The descriptor Kier and Hall index (order 2) shows significant negative correlation with logIC_50_ (R = −0.560), indicating a reverse relation.

The best model related to TrkA had architecture 5-5-4-1 and included the largest numbers of training and validation data points (N_tr_ = 121, N_val_ = 30). The model was trained up to 1641 epochs and resulted in RMS of 0.216 and 0.230 for the training and validation set, respectively (see Table 1 and Figure 1C, Supplementary Table S3). This model obtained the highest regression fit (R^2^_tr_ = 0.781, R^2^_val_ = 0.798) compared to the other models for the validation set. This maybe due to the lowest statistical variability of the training values logIC_50_ [−0.346, 3.076] and less structural diversity of the set. Regarding the input descriptors for the TrkA model, they can be attributed to (i) stability of the compound (lowest total interaction (AM1) for N–H bonds, lowest n-n repulsion (AM1) for C–N bonds, relative number of aromatic bonds); and (ii) hydrogen bonding acceptor ability of the molecule (HASA-2/TMSA—Hydrogen Acceptor Surface Area type 2/Total Molecular Solvent Area) (AM1), square root of partial charged (AM1) surface area of C atoms). According to the descriptor Lowest total interaction (AM1) for N–H bonds, larger values contribute to large logIC_50_ values (R = 0.655). There are eight compounds clustered in the highest range of logIC_50_, as can be seen from Figure 1C. These compounds are characterized with zero values of lowest total interaction (AM1) for N–H bonds, lowest n-n repulsion (AM1) for C–N bonds, and relative number of aromatic bonds.

### 2.2. Linear QSAR

**Figure 2:**
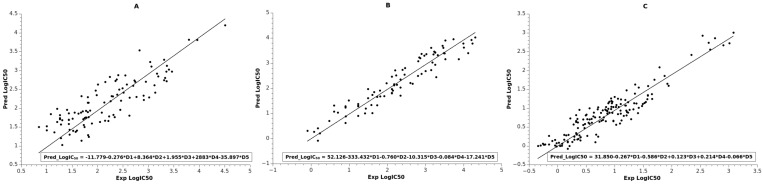
Linear fit between experimental and observed logIC_50_ of the BMLR models: (**A**) LRRK2 set; (**B**) NMDA set; (**C**) TrkA set. Trend lines indicated by straight lines. BMLR equations in Table 2 are also incorporated in the boxes.

The best multilinear regression (BMLR) algorithm was used to generate several multilinear equations for the dataset with between two and seven descriptors. The best models for each target are presented in Table 2, with their statistical parameters and descriptors. The final model for LRRK2 set had five descriptors. As can be seen from Table 2, the quality of linear equation resulted in R^2^ = 0.721 and average ABC validation R^2^_pred_ = 0.725 with Fisher’s statistic F = 49. The plot between predicted and observed logIC_50_ is shown in Figure 2A, and the values are collected in Supplementary Table S1. The most statistically significant descriptor in the model according to the *t*-test is Highest coulombic interaction (AM1), which is related to the stability of the compound and its electrostatic interactions. The other descriptor related to the stability of the compound is Maximum bonding contribution of one MO (AM1). The remaining descriptors are related to the reactivity of the compound (Maximum electrophilic reactivity index (AM1) for H atoms, Maximum nucleophilic reactivity index (AM1) for C atoms), and its electrostatic interaction is confined within the negative charge (Relative negative charge—RNCG (QMNEG/QTMINUS) (Zefirov)). It should be noted that the descriptors for this model are similar to the descriptors in the ANN model for LRRK2 (see Table 1).

The next BMLR model for the NMDA set resulted in five descriptors (Table 2). However, in this exercise the full set of 133 data points was not used as it was in the case of the ANN model for NMDA. The reason was that linear modeling did not succeed in producing a statistically significant equation. We then performed leverage analysis of the initial models in order to reduce the number of data points for the BMLR to 83. Most of the reduced compounds contained Br or/and Cl atoms. Thus, the final model had significant quality of fit R^2^ = 0.906, as shown in Figure 2B (Supplementary Table S2), with Fisher’s statistic F = 149 and average ABC prediction of R^2^_pred_ = 0.901. The most statistically significant descriptor in the model is the Kier and Hall index, which, together with the descriptor Average bonding information content (order 0), indicates the branching and compactness of the compounds. The Kier and Hall index descriptor also appears in the corresponding ANN model and with the same trend as discussed in Section 2.1. The remaining descriptors can be attributed to the hydrogen acceptor ability (HACA-2/TMSA) and stability (Average valency (AM1) for C atoms, Lowest e-n attraction (AM1) for C–N bonds) of the compounds.

The final BMLR model for the TrkA set resulted in an equation also containing five descriptors, as indicated in Table 2. The model coefficient of determination was R^2^ = 0.866, with Fisher’s statistic F = 187 and average ABC validation R^2^_pred_ = 0.861 (see Figure 2C; values for the plot in Supplementary Table S3). It should be noted that this BMLR model had better quality of linear fit compared to the corresponding ANN model (R^2^_tr_ = 0.781) for the TrkA data. This might be attributed to the superior feature selection of the BMLR model as compared to the ANN descriptor selection. The most statistically significant descriptor in the equation is Highest coulombic interaction (AM1) for N–H bonds, and therefore it is related to the stability of the N–H bonds. The next most significant descriptor in the equation is Number of F atoms. This descriptor reflects the importance of the F atoms, as more than 90% of the compounds in the set include F atoms. The remaining descriptors are related to the electrostatic interactions and stability of the compounds (Charged (Zefirov) surface area of N atoms, Total point-charge component of the molecular dipole (AM1), Highest e-n attraction (AM1) for C–C bonds).

### 2.3. Virtual Screening of Natural Compounds

In this exploration, we used the models developed in Section 2.1 and Section 2.2 to predict logIC_50_ values of 13,648 natural compounds extracted from the ZINC database [38]. The compounds were in ascending order according to their predicted logIC_50_ by both ANN and BMLR models. In this way, the top 100 compounds were further selected by average prediction by both models. During prediction of the compounds, we also used the model’s applicability domain, described in Section 3.5. The resulting three sets of compounds were submitted for molecular docking and molecular dynamics analysis.

### 2.4. Molecular Modeling Results

The top 40% of compounds predicted by the QSAR models (see Section 2.3) for each biological target were further examined using the molecular modeling techniques. Eighty compounds for NMDA and LRRK2 and 40 for TrkA were studied by molecular docking. In each case, molecular dynamics simulations were further carried out for the three best compounds. The results were compared with the modeling data on known NMDA, LRRK2, and TrkA inhibitors (see Table 3).

#### 2.4.1. NMDA

**Figure 3:**
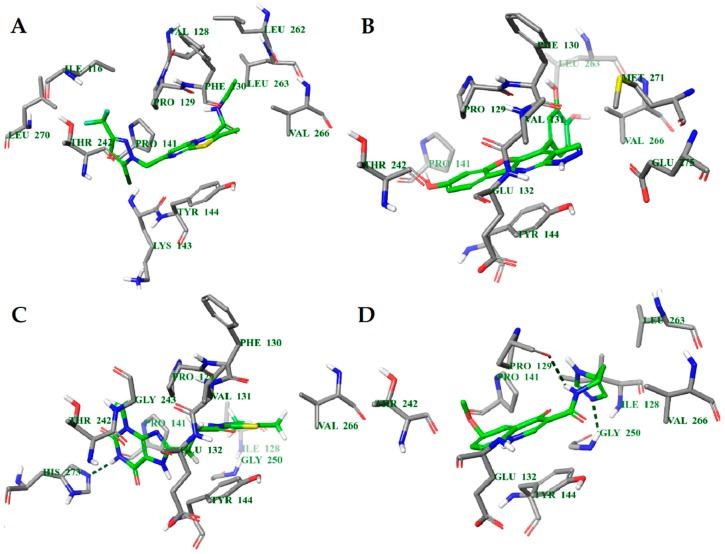
Calculated binding modes of ligands in the active site of NMDA (PDB ID: 5TP9): (**A**) compound GNE-5729, (**B**) compound **1N**, (**C**) compound **2N**, (**D**) compound **3N**. The amino acid residues of NMDA are colored gray (carbon), blue (nitrogen), red (oxygen), and white (hydrogen). Hydrogen bonds formed between compounds and residues of NMDA are represented by green dashed lines.

The molecular docking binding energies of the selected compounds span the interval of −11.9 to −2.2 kcal/mol and the ligand efficiencies are between −0.40 and −0.02. The best three compounds by ligand efficiency, compounds **1N**, **2N**, and **3N**, have somewhat smaller binding energies (−9.8, −9.7, and −9.0 kcal/mol, respectively), but similar ligand efficiencies (−0.39, −0.40, and −0.39) compared to those for the known inhibitor GNE-5279 (−11.3 and −0.42 kcal/mol). The binding modes of the three predicted compounds and of the inhibitor GNE-5279 are given in Figure 3.

In order to elaborate the ligand–enzyme interactions further, molecular dynamics simulations of 50 ns were carried out for all four compounds. The root mean standard deviation (RMSD) of the ligand and protein was stable between 1 and 4 Å, except for compound **2N** which had change at about 35 ns (see Supplementary Figure S1). Therefore, only the first 30 ns was taken into account in further data analysis for this compound.

**Figure 4:**
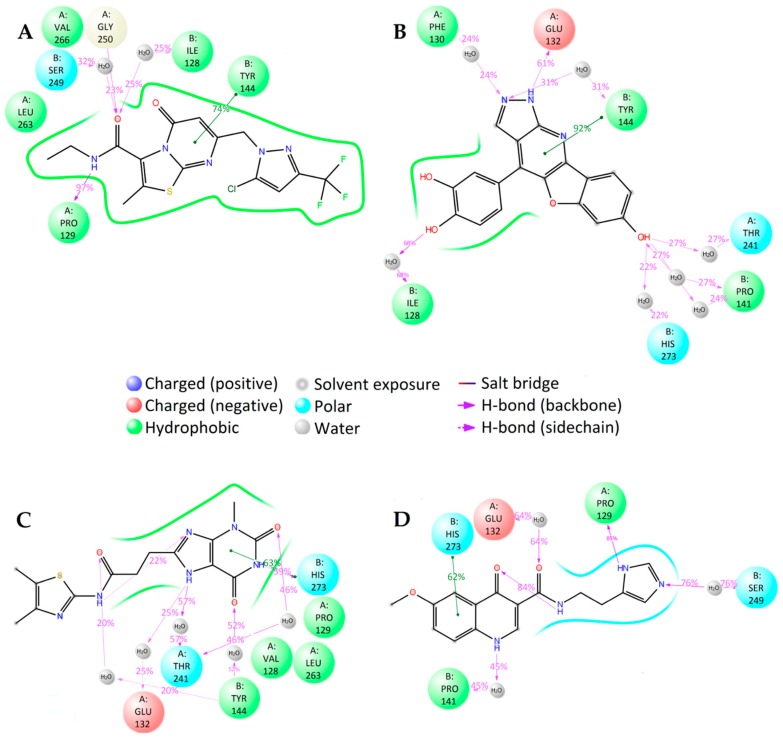
2D summary of molecular dynamics calculated contacts between NMDA and compounds (**A**) GNE-5729, (**B**) **1N**, (**C**) **2N**, and (**D**) **3N**.

There are notable differences in the calculated binding of ligand compounds to NMDA. The molecular dynamics calculated contacts of compound **3N** are similar to known inhibitor GNE-5279 (Figure 4A,D and Supplementary Figure S2A,D), involving strong hydrogen bonding with PRO129 and hydrophobic interactions around TYR144 of the protein. The binding pictures for compounds **1N** and **2N** are very different, being directed primarily by hydrophobic interactions and bonding through water molecules (Figure 4B,C and Supplementary Figure S2B,C).

The interactions between the ligands and the NMDA protein were also analyzed with the MM-GBSA method (Supplementary Table S4). The total binding energy for the studied compounds is smaller than that of the known NMDA inhibitor GNE-5279. Nevertheless, the ligand efficiency for compound **2N** is still too high to suggest it as a potential new inhibitor.

#### 2.4.2. LRRK2

**Figure 5:**
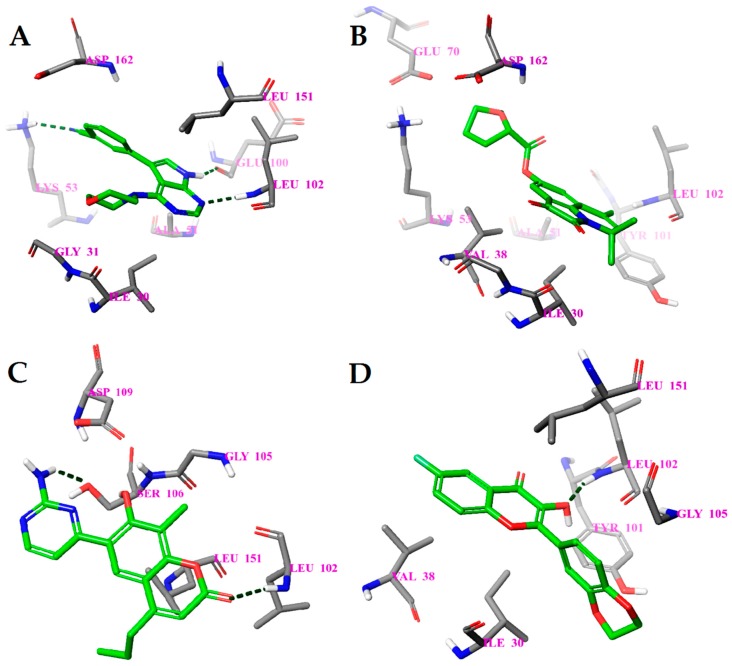
Calculated binding modes of (**A**) compound PF-06447475, (**B**) compound **1L**, (**C**) compound **2L**, and (**D**) compound **3L** in the active site of LRRK2 (PDB ID: 4U8Z). The amino acid residues of LRRK2 are colored gray (carbon), blue (nitrogen), red (oxygen), and white (hydrogen). Hydrogen bonds formed between compound and residues of LRRK2 are represented by green dashed lines.

The binding energies of the compounds selected from ANN results for molecular docking were between −9.7 and −5.9 kcal/mol and the respective ligand efficiencies in the interval −0.38 to −0.17. The best three compounds by ligand efficiency, compounds **1L**, **2L**, and **3L**, have very similar binding energies (−9.0, −8.5, and −8.7 kcal/mol, respectively) and ligand efficiencies (−0.36, −0.37, and −0.38, respectively) compared to those for the known inhibitor PF-06447475 (−9.0 and −0.39 kcal/mol, respectively) (Table 3). The binding modes of these three compounds and the inhibitor PF-06447475 are given in Figure 5. Again, molecular dynamics simulations of 50 ns were carried out for all four compounds. The root mean standard deviation (RMSD) of the ligand and protein was stable between 0.8 and 3.6 Å for all compounds (Supplementary Figure S3). To examine the stability of the molecular dynamics simulations in time, we carried out additional runs of 20, 40, and 60 ns for the ligand **1L** (see Supplementary Figures S5 and S6). The RMSDs of ligand and protein positions and binding histograms obtained by multiple runs demonstrate the stability of the simulations and congruency of the results.

**Figure 6:**
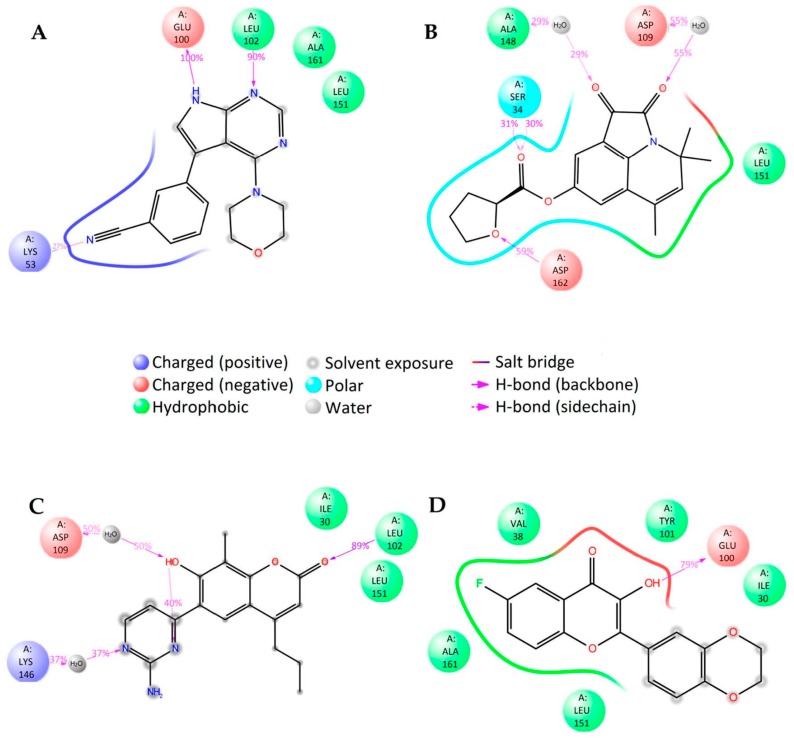
2D summary of the molecular dynamics calculated contacts between compounds (**A**) PF-06447475, (**B**) **1L**, (**C**) **2L**, (**D**) **3L** and LRRK2.

In the case of LRRK2, molecular dynamics simulations indicate some similarities in the binding of different ligands. The molecular dynamics calculated contacts of compounds **2L** and **3L** include strong hydrogen bonding between ligand and peptide links at amino acid residues GLU100 and/or LEU102, similarly to known inhibitor PF-06447475 (Figure 6A,C,D and Supplementary Figure S4A,C,D). The molecular dynamics simulated binding picture of compound **1L** is different, with two relatively strong hydrogen bonds at the SER34 and ASP162 residues of the LRRK2 protein (Figure 6B and Supplementary Figure S4B). Nevertheless, as it is located in the active site of the enzyme, this compound may also act as an inhibitor. Furthermore, when the interactions between the ligands and the LRRK2 protein were calculated using the MM-GBSA method (Supplementary Table S5), the compound **1L** gave significantly better interaction energy (−100.04 kcal/mol) and ligand efficiency (−4.00) than the known inhibitor PF-06447475 (−72.41 kcal/mol and −3.15, respectively). Consequently, this compound (9,11,11-trimethyl-2,3-dioxo-1-azatricyclo[6.3.1.04^4^,^12^]dodeca-4(12),5,7,9-tetraen-6-yloxolane-2-carboxylate) is predicted as a potential new strong LRRK2 inhibitor.

#### 2.4.3. TrkA

**Figure 7:**
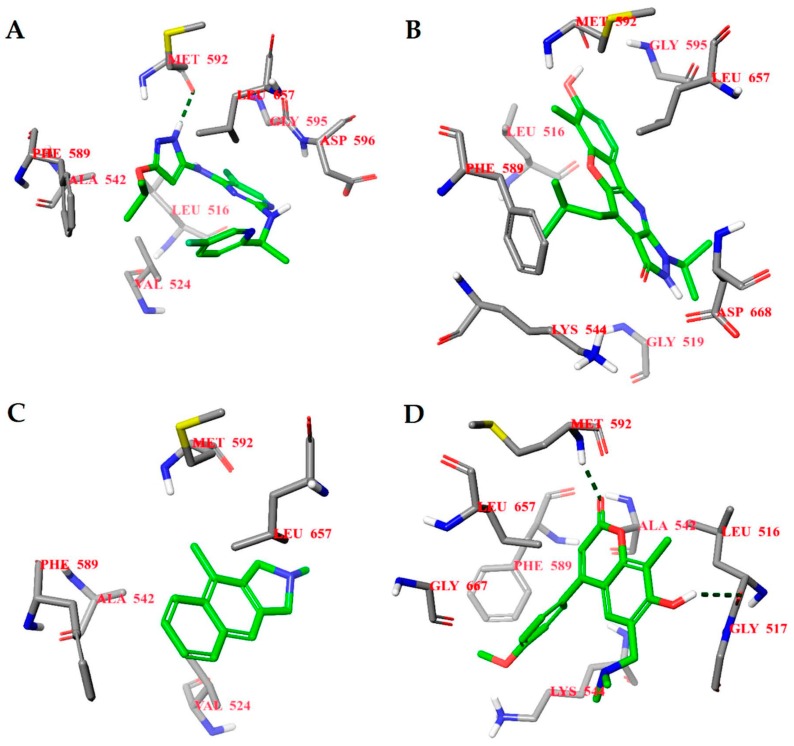
Calculated binding modes of (**A**) compound AZ-23, (**B**) compound **1T**, (**C**) compound **2T**, and (**D**) compound **3T** in the active site of TrkA (PDB ID: 4AOJ). The amino acid residues of TrkA are colored gray (carbon), blue (nitrogen), red (oxygen), and white (hydrogen). Hydrogen bonds formed between compound and residues of TrkA are represented by green dashed lines.

The binding energies of the 40 compounds selected from ANN results for molecular docking were between −11.9 and −3.5 kcal/mol and the respective ligand efficiencies in the interval −0.5 to −0.16. The best three compounds (**1T**, **2T**, and **3T**) had significantly better ligand efficiency than the known TrkA inhibitor AZ-23 (Table 1). In particular, compound **2T**, with almost the same binding energy as compound AZ-23 (−8.6 vs. −8.7 kcal mol), had much higher ligand efficiency (−0.54 vs. −0.32). The binding modes of the three selected best inhibitor candidates and the inhibitor AZ-23 are given in Figure 7. Molecular dynamics simulations of 50 ns were also carried out for all four compounds. The observed root mean standard deviations (RMSDs) of the ligand and protein were stable between 1 and 4.5 Å for all compounds, indicating the stability of the respective complexes (Supplementary Figure S7).

**Figure 8:**
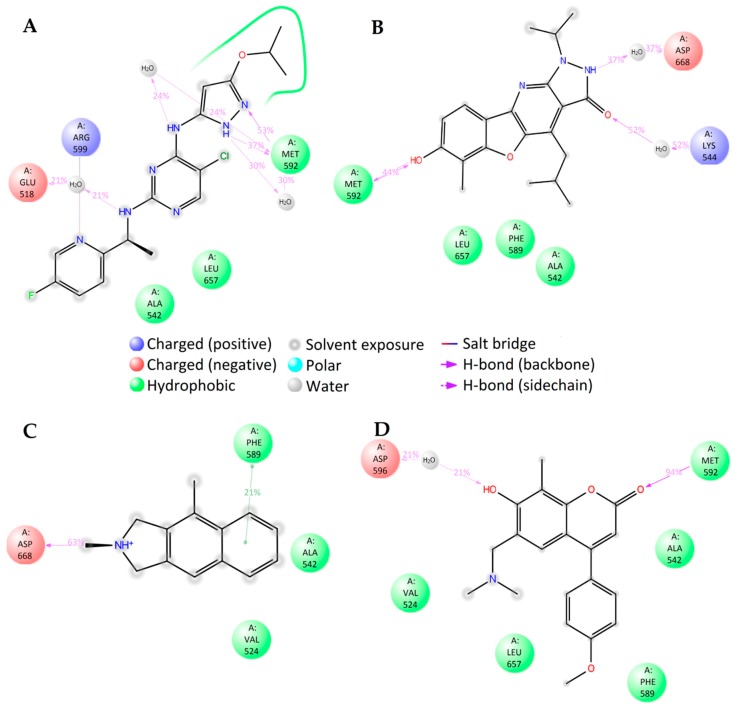
2D summary of molecular dynamics calculated contacts between compounds (**A**) AZ-23, (**B**) **1T**, (**C**) **2T**, and (**D**) **3T** and TrkA.

The molecular dynamics simulations of TrkA with different ligands show close similarity in the binding of compounds **1T**, **3T**, and known inhibitor compound AZ-23. The main directing interactions are the strong hydrogen bonding at amino acid residue MET592 and hydrophobic interactions around PHE589, LEU657, and VAL524 residues (Figure 8A,B,D and Supplementary Figure S8A,B,D). The binding mode of positively charged compound **1T** was very different, as expected, involving the hydrogen bonding between the NH+ group of the ligand and the ASP668 residue of the protein (Figure 7C and Supplementary Figure S8C).

An analysis of the interactions between the studied ligands and the TrkA protein using the MM-GBSA method (Supplementary Table S6) gives close ligand efficiencies for compounds **2T** and **3T** and inhibitor AZ-23 (−2.69, −2.79, and −2.61, respectively). Thus these two compounds can be tested as potential new TrkA inhibitors.

## 3. Materials and Methods 

### 3.1. Data for Building QSAR Models

The data for building the current QSAR models were extracted from the ChEMBL database [39]. The measured inhibitory concentration IC_50_[nM] (or log IC_50_) was used as a dependent variable in all QSAR models. Several criteria were applied for the preparation of the three datasets related to TrkA, NMDA, and LRRK2 proteins: (i) where possible, newer experimental data were preferable; (ii) preferably the same (or similar) experimental protocol for activity measurements was used; (iii) where possible, data were obtained by a single laboratory, author, or group; (iv) diverse structural compounds with low molecular weight were used; and (v) statistical range of the measured activity for each set was a minimum 2 to 3 logarithmic units. Thus, 151, 133, and 101 compound datasets were prepared for TrkA, NMDA, and LRRK2, respectively. The data collected are shown in Supplementary Tables S1, S2 and S3).

### 3.2. Geometry Optimization and Descriptor Generation

The two-dimensional molecular structures of the compounds obtained in Section 3.1 were converted into three-dimensional structures using Open Babel [40]. Conformational search was carried out by the CMol3D program of FQSARModel [41,42] for the dataset structures, where random conformations were constructed by means of the stochastic proximity embedding algorithm [43], followed by optimization based on MMFF94s force field [44] to improve their quality. Thereafter, all geometries were optimized as random vacuum conformer with the minimum potential energy using MOPAC 6.0 [45]. The quantum-mechanical semiempirical calculation in the form of AM1 [46] energy minimization was subsequently applied with a gradient of 0.01 kcal/Å as a stop criterion. The following mopac keywords were used for the optimization procedure: AM1, VECTORS, BONDS, PI, POLAR, PRECISE, ENPART, EF, MMOK, NOINTER, GNORM = 0.05, XYZ.

For a given compound structure, it was possible to generate a large number (>600) of molecular descriptors [47] using the descriptor calculator in the FQSARModel program applied on the 3D structures obtained by MOPAC6. These features can be generally classified into 2 categories: structural and quantum-chemical descriptors. The first group is further divided into constitutional, topological, geometric, and electrostatic subgroups. The constitutional descriptors are simple fragment additive and mostly reflect the general properties of the compound’s structure. The topological descriptors were calculated using graph theory applied to the scheme of atom connections of the structure. The second main group included electronic and quantum chemical classes of descriptors. The best few descriptors found in Section 3.3 were used as independent variable inputs for the developed models.

### 3.3. Development of QSAR Models

#### 3.3.1. ANN Modeling

In the present study, we developed a fully connected neural network with a back-propagation algorithm of the error [48]. The ANNs constructed were used in the building of the nonlinear models for all activities. Training of the net (optimization of the weights) was performed by generalized delta rule. A hyperbolic activation function was applied for the neurons’ transformations. In order to find important descriptors as inputs to the net, a sensitivity analysis was performed on a preselected descriptor space, based on the lowest root mean squared error (RMS) or Pearson’s correlation coefficient (R) with respect to logIC_50_. The descriptor space was formed after applying the following criteria to reduce the total descriptor space: (i) all descriptors with variance less than 10^−4^ were excluded, (ii) descriptors that did not have indicated Pearson correlation coefficient R > 0.2 with respect to the property were excluded, and (iii) certain chemically irrelevant descriptors were inspected. Further, all the remaining descriptors were correlated with the property in order to extract (and use as inputs to the net) the best few with highest coefficient of determination (R^2^). Prior to this procedure, the descriptors were normalized according to their variation (distance between minimum and maximum values) and standard deviation. The main reason for such selection is that the descriptors can be explained by/related to the mechanistic picture behind the property interaction in a way similar to the multilinear regression models. For instance, a positive correlation in MLR would suggest that with increased descriptor value, the property value would also increase.

To find an optimal ANN architecture, we followed the common principle of generality of ANN prediction [49] i.e., seek the lowest possible number of neurons for the smallest structure. Several ANN models with different architectures were built for each logIC_50_ related to the different training sets for the NMDA, TrkA, and LRRK2 proteins. In addition, we monitored the RMS (or Pearson’s correlation coefficient R, or R^2^) for each architecture (regarding the hidden units in the hidden layer). This procedure was done in order to select the topology with the lowest RMS (highest R). The number of layers was chosen to be three- to fourfold based on common practice (usually not more than 2 hidden layers) for the QSAR ANN modeling and by taking into account the number of data points to reduce the chance of overfitting during the training stage. The whole ANN training procedure was performed by the ANN module in FQSARModel.

Validation of the ANN models was carried out by using training and validation (selection) sets. These subsets were constructed to reflect the distribution of the experimental property values of the whole dataset. The chosen validation sets included each third or fifth data point of the total data in a set (see Section 3.1, Supplementary S1). In this way, the validation set was used to train the network to avoid overfitting by stopping the training procedure prematurely when the RMS_val_ started to increase.

#### 3.3.2. MLR Modeling

The best multilinear regression (BMLR) method [50] was used to find the best correlation models from selected non-collinear descriptors. This approach gradually builds multiple regression equations by searching the first few descriptors among a large descriptor space, such as that obtained in Section 3.2. Thus, BMLR selects the best 2-parameter regression equations, the best 3-parameter regression equations, etc., based on the highest R^2^ and F values in a stepwise regression procedure. The result obtained by BMLR is the “best” linear representation of the activity (logIC_50_) in a given descriptor pool.

An ABC validation test [51] was applied to estimate the predictivity of the MLR equations developed, taking into account the property data distribution. The ABC method consists of sorting the data in ascending order according to the observed (experimental) values and forming 3 subsets (A, B, C): the first, fourth, seventh, etc., data points comprised the first subset, A; the second, fifth, eighth, etc., comprised the second subset, B; and the third, sixth, ninth, etc., comprised the third subset, C. Then the three training sets were prepared as combinations of any two subsets. Subsequently, the tested MLR model was rebuilt for each of the training sets (AB, AC, and BC), with the same descriptors but with other optimized regression coefficients. Further, these three models, AB, AC, and BC, were used to predict the property values for the C, B, and A subsets, respectively. The prediction was assessed based on the coefficient of determination of R^2^ between the predicted and observed property values. The final result was estimated by the average square correlation coefficient by the three “external” sets C, B, and A, R^2^_pred_. If the average R^2^_pred_ is close to the R^2^ of the model, it indicates satisfactory prediction of that model. In addition to the ABC validation, the standard leave-one-out cross-validation (R^2^_cv_) for the MLR model was also used.

### 3.4. Molecular Modeling

#### 3.4.1. Targets

In our study we used the LRRK2 homology model MST3 crystal structure, which was obtained from the Protein Data Bank (PDB) (ID: 4U8Z) [52]. The structural model was measured by X-ray diffraction with resolution 1.63 Å. The structure of the human GluN1/GluN2A ligand-binding domain (LBD) was obtained from the Protein Data Bank (ID: 5TP9). The crystal structure of LBD of NMDA was measured by X-ray diffraction with resolution 2.4 Å [53]. The crystal structure of TrkA was obtained from the Protein Data Bank (ID: 4AOJ), with resolution 2.75 Å measured by X-ray diffraction. This structure is actually a trimer of TrkA, with individual protein molecules denoted as chain A, chain B, and chain C. For modeling purposes, only a single TrkA molecule is needed, thus just chain A was used [54]. Raw crystal structures were corrected and hydrogen atoms were automatically added to the protein using Schrödinger’s Protein Preparation Wizard of Maestro 10.7 [55,56,57]. Water molecules were removed from the crystal structure.

Small molecule structures. The two-dimensional chemical structures of ligands were downloaded using the ZINC [38] tool and the database. For ligand structure preparation we used Ligprep from the Schrödinger suite [58]. Ligprep used the OPLS2005 force field in all ligand preparation steps. Generation of all possible states and ionization states was enumerated for each ligand using Epik at a pH of 7.0 ± 2. Stereoisomers were determined from 3D structure. PDB files for the molecular docking procedure were created from lowest energy conformers for each ligand.

#### 3.4.2. Molecular Docking

AutoDock Vina 1.1.2 [59] was used for the docking studies to determine binding modes and binding energies of ligands to the receptor. Schrödinger’s Glide Grid Generation was used to identify the binding interface between the co-crystallized ligand and receptor for each structure [60]. The active site was surrounded by a grid box with a size of 20 × 20 × 20 points and spacing of 1.000 Å. The settings used for the iterated local search global optimizer, based on mutation and local optimization steps accepted or rejected with a Metropolis criterion in Vina, were 9 modes, 1 central processing unit, and energy range of 1 kcal/mol. Other settings were used as default.

#### 3.4.3. Molecular Dynamics

The molecular dynamic simulations were carried out using the Desmond simulation package of Schrödinger [61]. In all runs, the NPT (isothermal–isobaric) ensemble was applied with a temperature of 300 K and pressure of 1 bar. The simulation length was 50 ns, with relaxation time 1 ps. The force field parameters for each simulation were according to OPLS_2005 [62]. The long-range electrostatic interactions were calculated using the particle mesh Ewald (PME) method [63]. The cutoff radius in Coloumb interactions was 9.0 Å. The water molecules were described using a simple point charge model (SPC) [64]. The Martyna–Tuckerman–Klein chain coupling scheme [65] with a coupling constant of 2.0 ps was used for pressure control and the Nosé–Hoover chain coupling scheme for temperature control. Nonbonded forces were calculated using an r-RESPA integrator, where the short-range forces were updated every step and the long-range forces were updated every 3 steps. The trajectories were saved at 4.8 ps intervals for analysis. To analyze the behavior and interactions between the ligands and protein, we used the Simulation Interactions Diagram tool implemented in the Desmond molecular dynamics package.

### 3.5. Virtual Screening of Database of Natural Compounds

In our study, we screened the ZINC [38] database for potential natural agonists/antagonists by utilizing the nonlinear and linear QSAR models developed in Section 2. We extracted nearly 17,000 compounds collected in the natural product database subsection of ZINC. The collections were attributed to AnalytiCon Discovery and IBScreen providers [66,67] within ZINC sets. We further refined the NPs by applying general criteria as follows: (i) removal of duplicates, (ii) removal of compounds with unclear structural connectivity, (iii) removal of compounds with molecular weight >600 amu, and (iv) removal of compounds without purchasable information. In this way, the number of NPs was reduced to 13,648. These compounds were further submitted to geometric optimization and descriptor calculation as described in Section 3.2. With the availability of the molecular descriptors, the ANN and MLR QSAR models were then employed to predict NPs with low inhibitory concentration. However, it is important for a predictive QSAR model that certain limits are defined for future predictions of compounds, i.e., applicability domain (AD). We defined the applicability domain of the general ANN and BMLR models quantitatively, proceeding from the minimum and maximum descriptor values for the corresponding training sets in Section 3.1. Our practice showed that predictions of new external compounds (with descriptor D_ix_) are reasonable to be bound within the descriptor interval [D_imin_,D_imax_] augmented by ±|D_imax_ − D_imin_| × 0.3, where D_imin_,D_imax_ are the minimum and maximum descriptor values for the training set for the *i*th descriptor (shown in square brackets above). This condition has to be simultaneously fulfilled for all D_ix_ descriptors so that the QSAR models give realistic predictions.

## 4. Conclusions

In this study we have identified several potential candidates for further research on drugs to treat neurodegenerative diseases related to LRRK2, NMDA, and TrkA proteins. These inhibitors are based on natural products and have passed through a full cycle of in silico research from QSAR modeling through virtual screening, molecular docking, and molecular dynamics. We have also demonstrated that NPs are still a viable source of drug research despite the recent decline in this area.

The topical scientific literature indicates that there has been little exploration of NPs as potential drug candidates for neurodegenerative diseases using hybrid combinations of in silico techniques. This is especially true for CADD related to protein targets such as NMDA, LRRK2, and TrkA and small natural ligands. There are, however, many studies related to the above diseases and in silico modeling, but they do not concentrate on finding hit compounds in the NP space. Therefore, we also showed here that in silico techniques can be applied in the large NP space and can obtain potential hits quickly. A similar work by Ambure et al. [35] also indicated that this type of in silico modeling could lead to fast and reliable results. Their study was based on a classification QSAR problem, while our modeling is based on a regression problem. In this way, our QSAR models could predict more precisely in the range of IC_50_ values as compared to Ambure et al.’s classification models. Notably, our approach enabled to detect new scaffolds of the compounds structure. The chemical modification of compounds based on these scaffolds can lead to novel drug candidates against the pharmacological targets studied. A natural continuation of the present work, therefore, is experimental confirmation (in vitro/in vivo) of the best hit candidates found herein and their further structural optimization.

## Figures and Tables

**Table 1:** Statistical and topological parameters of the artificial neural network (ANN) models. LRRK2, leucine-rich repeat kinase 2; NMDA, *N*-methyl-d-aspartate; TrkA, tropomyosin *receptor* kinase A; RMS, root mean squared error.

Target	Alpha	Eta	N_tr_	N_val_	Epoch	R^2^_tr_	R^2^_val_	RMS_tr_	RMS_val_	Architecture	Input Descriptors
**LRRK2**	0.01	0.02	81	20	3525	0.788	0.565	0.195	0.381	5-5-3-1	Highest coulombic interaction (AM1); HPSA polar (AM1) part of SASA; max nucleophilic reactivity index (AM1) for C atoms; avg. electrophilic reactivity index (AM1) for C atoms; max electrophilic reactivity index (AM1) for N atoms
**NMDA**	0.01	0.02	107	26	1651	0.752	0.519	0.273	0.444	5-5-5-1	Kier and Hall index (order 2); charged (Zefirov) surface area of O atoms; min nucleophilic reactivity index (AM1) for O atoms; highest e-e repulsion (AM1) for C–O bonds; square root of charged (Zefirov) surface area of N atoms
**TrkA**	0.01	0.03	121	30	1641	0.781	0.798	0.216	0.230	5-5-4-1	Lowest total interaction (AM1) for N–H bonds; relative number of aromatic bonds; HASA-2/TMSA (AM1); square root of partial charged (AM1) surface area of C atoms; lowest n-n repulsion (AM1) for C–N bonds

**Table 2:** Best multilinear regression (BMLR) models for logIC_50_ and their statistical parameters.

**LRRK2**	**N = 101, R^2^ = 0.721, R^2^_cv_ = 0.683, R^2^_abc_ = 0.725, s^2^ = 0.167, F = 49.045**				
	**No**	**B**	**Errors B**	***t*-Statistics**	**Descriptor (D_i_)**
	0	−11.779	2.126	−5.541	Intercept
	1	−0.276	0.028	−9.947	Highest coulombic interaction (AM1)
	2	8.364	1.087	7.697	Max bonding contribution of one MO (AM1)
	3	1.955	0.954	2.048	RNCG relative negative charge (QMNEG/QTMINUS) (Zefirov)
	4	2883.443	389.061	7.411	Max electrophilic reactivity index (AM1) for H atoms
	5	−35.897	8.661	−4.145	Max nucleophilic reactivity index (AM1) for C atoms
	**ABC Validation**				
			(AB,C): R^2^_ab_ = 0.673	R^2^_ab_cv_ = 0.607	R^2^_c_pred_ = 0.729
			(BC,A): R^2^_bc_ = 0.722	R^2^_bc_cv_ = 0.662	R^2^_a_pred_ = 0.756
			(CA,B): R^2^_ca_ = 0.677	R^2^_ca_cv_ = 0.567	R^2^_b_pred_ = 0.689
**NMDA**	**N = 83, R^2^ = 0.906, R^2^_cv_ = 0.893, R^2^_abc_ = 0.901, s^2^ = 0.123, F = 149.268**				
	0	52.126	11.055	4.715	Intercept
	1	−333.432	34.324	−9.714	HACA-2/TMSA (Zefirov)
	2	−0.760	0.029	−26.281	Kier and Hall index (order 2)
	3	−10.315	0.785	−13.141	Average bonding information content (order 0)
	4	−0.084	0.008	−10.313	Lowest e-n attraction (AM1) for C–N bonds
	5	−17.241	3.022	−5.706	Average valency (AM1) for C atoms
	**ABC Validation**				
			(AB,C): R^2^_ab_ = 0.905	R^2^_ab_cv_ = 0.883	R^2^_c_pred_ = 0.894
			(BC,A): R^2^_bc_ = 0.920	R^2^_bc_cv_ = 0.900	R^2^_a_pred_ = 0.884
			(CA,B): R^2^_ca_ = 0.888	R^2^_ca_cv_ = 0.860	R^2^_b_pred_ = 0.926
**TrkA**	**N = 151, R^2^ = 0.866, R^2^_cv_ = 0.855, R^2^_abc_ = 0.861, s^2^ = 0.065, F = 187.251**				
	0	31.850	2.351	13.545	Intercept
	1	−0.267	0.017	−15.942	Highest coulombic interaction (AM1) for N–H bonds
	2	−0.586	0.041	−14.412	Number of F atoms
	3	0.123	0.010	12.637	Highest e-n attraction (AM1) for C–C bonds
	4	0.214	0.021	10.270	Charged (Zefirov) surface area of N atoms
	5	−0.066	0.017	−3.915	Total point-charge component of molecular dipole (AM1)
	**ABC Validation**				
			(AB,C): R^2^_ab_ = 0.859	R^2^_ab_cv_ = 0.842	R^2^_c_pred_ = 0.880
			(BC,A): R^2^_bc_ = 0.854	R^2^_bc_cv_ = 0.837	R^2^_a_pred_ = 0.861
			(CA,B): R^2^_ac_ = 0.861	R^2^_ac_cv_ = 0.841	R^2^_b_pred_ = 0.843

**Table 3:** Binding energies (kcal/mol) and binding modes of small-molecule ligands to receptors (LRRK2, NMDA, and TrkA).

No.	Structure	ZINC Code	Binding Energy ΔG, kcal/mol	Ligand Efficiency	Binding Mode, Including H-Bonds (Residue of Amino Acid Group or Atom in a Compound)
	**NMDA**				
GNE-5729	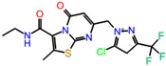	====	−11.3	−0.42	Ile116, Val128, Pro129, Phe130, Pro141, Lys143, Tyr144, Thr242, Leu262, Leu263, Val266, Leu270
**1N**	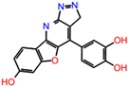	ZINC98363705	−9.8	−0.39	Pro129, Phe130, Val131, Glu132, Pro141, Tyr144, Leu263, Val266, Met271, Glu275
2N	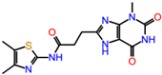	ZINC67658347	−9.7	−0.40	Ile128, Pro129, Val131, Glu132, Pro141, Tyr144, Thr242, Gly243, Gly250, Val266, His273 (N...HN)
**3N**	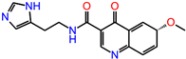	ZINC98364250	−9.0	−0.39	Ile128, Pro129, Glu132, Pro141, Tyr144, Thr242, Gly250 (NH...N), Leu263, Val266
**LRRK2**					
PF-06447475	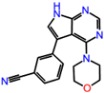	====	−9.0	−0.39	Ile30, Glu31, Ala51, Lys53, Glu100 (O...HN), Leu102 (NH...N), Leu151, Asp162
**1L**	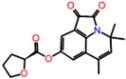	ZINC2115150	−9.0	−0.36	Ile30, Val38, Lys53, Glu70, Tyr101, Leu102, Asp162
**2L**	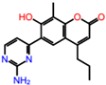	ZINC12901845	−8.5	−0.37	Leu102 (NH...O), Ser106 (HO....H2N), Gly105, Asp109, Leu151
**3L**	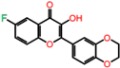	ZINC518729	−8.7	−0.38	Ile30, Val38, Tyr101, Leu102 (NH...OH), Gly105, Leu151
TrkA					
**AZ-23**	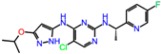	ZINC35077985	−8.7	−0.32	Leu516, Val524, Ala542, Phe589, Met592, Gly595, Asp596, Leu657 (O....HN)
**1T**	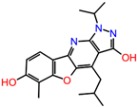	ZINC85880015	−9.80	−0.38	Leu516, Gly519, Lys544, Phe589, Met592, Gly595, Leu657, Asp668
**2T**	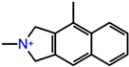	ZINC1323099	−8.6	−0.54	Val524, Ala542, Phe589, Met592, Leu657
**3T**	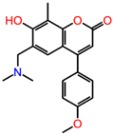	ZINC489632	−9.5	−0.38	*Leu516* (*O... HO*), Gly517, Ala542, Lys544, Phe589, Met592 (NH...O), Leu657, Gly667

## Supplementary Materials

The following are available online at http://www.mdpi.com/1420-3049/23/8/1847/s1: Table S1. LRRK2 dataset. Predicted and experimental logIC_50_ values for ANN and BMLR models. Table S2. NMDA dataset. Predicted and experimental logIC_50_ values for ANN and BMLR models. Table S3. TrkA dataset. Predicted and experimental logIC_50_ values for ANN and BMLR models. Table S4. Binding free energies (in kcal/mol) of NMDA—ligand complexes calculated using the MM/GBSA method. Table S5. Binding free energies (in kcal/mol) of LRRK2—ligand complexes calculated using the MM/GBSA method. Table S6. Binding free energies (in kcal/mol) of TrkA—ligand complexes calculated using the MM/GBSA method. Figure S1. RMSDs of atomic positions for the compounds (A) GNE-5729, (B) **1N**, (C) **2N**, and (D) **3N** (in red) and the receptor NMDA (in blue) of 50 ns molecular dynamics simulations using Desmond code. Figure S2. Molecular dynamics calculated contacts between compounds (A) GNE-5729, (B) **1N**, (C) **2N**, and (D) **3N** and NMDA. Figure S3. RMSDs of atomic positions for the compounds (A) PF-06447475, (B) **1L**, (C) **2L**, and (D) **3L** (in red) and the receptor LRRK2 (in blue) of 50 ns molecular dynamics simulations using Desmond code. Figure S4. Molecular dynamics calculated contacts between compounds (A) PF-06447475, (B) **1L**, (C) **2L**, and (D) **3L** and LRRK2. Figure S5. RMSD of the atomic positions for the compounds **1L** (in red) and the receptor LRRK2 (in blue) of the 20 ns (A), 40 ns (B) and 60 ns (C) molecular dynamics simulations using Desmond code. Figure S6. Molecular dynamics calculated contacts between compound **1L** and LRRK2 (A) −20 ns, (B) −40 ns, (C) −60 ns. Figure S7. RMSDs of atomic positions for the compounds (A) AZ-23, (B) **1T**, (C) **2T**, and (D) **3T** (in red) and the receptor TrkA (in blue) of 50 ns molecular dynamics simulations using Desmond code. Figure S8. Molecular dynamics calculated contacts between compounds (A) AZ-23, (B) **1T**, (C) **2T**, and (D) **3T** and TrkA.
