# Supplementary material for: Identification of Natural Compounds against Neurodegenerative Diseases Using In Silico Techniques
Source: Molecules. 2018 Jul 25;23(8):1847. doi: 10.3390/molecules23081847 (PMC6222649; doi:10.3390/molecules23081847)
Supplement: Supplementary file 1 [file molecules-23-01847-s001.pdf]

Supplimentary Material

Table S1. LRRK2 data set . Predicted and experimental LogIC$_{50}$ values for ANN and BMLR model

ChEMBL ID	ANN-Pred_LogIC50[nM]	BMLR-Pred_LogIC50[nM]	Exp_LogIC50[nM]
CHEMBL2333112	2.256	1.640	1.204
CHEMBL2333114	1.685	1.766	1.820
CHEMBL2333116	1.728	1.741	1.255
CHEMBL2333117	3.768	2.288	2.628
CHEMBL2333119	1.917	1.763	1.771
CHEMBL2333120	1.594	1.852	1.079
CHEMBL2333121	1.401	1.029	1.301
CHEMBL2333122	1.926	1.807	2.621
CHEMBL2333124	2.708	3.027	3.400
CHEMBL2333126	1.523	1.586	1.431
CHEMBL2333128	1.625	1.510	0.845
CHEMBL2333129	1.446	1.577	1.279
CHEMBL2333130	1.478	1.704	1.431
CHEMBL2333131	1.795	1.509	1.740
CHEMBL2333132	1.546	1.731	1.505
CHEMBL2333133	1.746	1.715	1.716
CHEMBL2333134	1.512	1.561	1.724
CHEMBL2333135	1.824	1.748	1.748
CHEMBL2333138	1.827	1.669	2.210
CHEMBL2333139	1.711	2.498	2.422
CHEMBL2347804	1.519	1.208	1.279
CHEMBL2347805	1.361	1.305	1.447
CHEMBL2347806	1.393	1.142	1.681
CHEMBL2347807	1.443	1.295	1.813
CHEMBL2347808	2.569	2.206	2.459
CHEMBL2347810	1.347	1.369	1.146
CHEMBL2347811	1.497	1.867	1.431
CHEMBL2347812	1.688	1.933	1.771
CHEMBL2347813	2.170	1.889	2.079
CHEMBL2347814	2.250	1.965	2.444

Table S1. Cont.

CHEMBL2347817	1.447	1.410	1.591
CHEMBL2347818	1.335	1.693	1.431
CHEMBL2347819	1.958	1.944	1.851
CHEMBL2347820	3.847	2.979	3.461
CHEMBL2347822	3.389	2.980	3.348
CHEMBL2347823	1.367	1.435	1.447
CHEMBL2347826	1.499	1.423	1.000
CHEMBL2347827	1.486	1.990	1.204
CHEMBL2348937	2.376	2.321	2.301
CHEMBL2348939	2.321	1.889	1.672
CHEMBL2348941	3.006	2.612	3.118
CHEMBL2348943	1.163	1.524	1.000
CHEMBL2348945	1.828	1.824	1.255
CHEMBL2348946	2.772	1.966	1.398
CHEMBL2348947	1.533	1.887	1.544
CHEMBL2348948	2.352	2.155	2.127
CHEMBL2348950	2.321	2.148	1.806
CHEMBL2348951	2.181	2.885	2.403
CHEMBL2348952	3.072	2.290	3.010
CHEMBL2348953	2.228	1.922	1.708
CHEMBL2348955	2.463	2.188	2.253
CHEMBL2348956	3.770	2.533	2.490
CHEMBL2348957	3.489	3.294	3.305
CHEMBL2348960	2.618	2.750	3.307
CHEMBL2348964	1.742	1.599	1.886
CHEMBL2348967	1.856	1.244	1.826
CHEMBL2348968	2.037	2.634	2.004
CHEMBL2348969	1.576	1.406	1.519
CHEMBL2348970	2.010	2.704	2.149
CHEMBL2348971	2.065	2.327	1.681
CHEMBL2348972	2.092	1.769	2.332
CHEMBL2348973	2.230	2.323	2.272
CHEMBL2348974	2.517	1.788	2.223
CHEMBL2348975	2.835	2.305	2.764
CHEMBL2348978	3.131	2.338	2.902
CHEMBL2348983	2.932	2.851	3.192
CHEMBL2349092	2.490	2.725	3.356
CHEMBL3326114	2.633	2.836	2.238
CHEMBL3326115	2.882	2.599	2.695
CHEMBL3326116	2.782	2.368	2.384
CHEMBL3326117	2.573	2.875	2.543
CHEMBL3326118	2.983	2.707	2.944
CHEMBL3326119	2.150	2.151	1.820

Table S1. *Cont.*

CHEMBL3326120	3.530	3.129	3.360
CHEMBL3326124	3.080	2.227	2.905
CHEMBL3326125	1.653	2.471	1.959
CHEMBL3326126	1.912	2.355	1.978
CHEMBL3326127	2.232	2.730	2.350
CHEMBL3326128	2.678	2.300	1.806
CHEMBL3326129	2.736	2.418	2.509
CHEMBL3326130	2.848	3.229	3.029
CHEMBL3326131	3.348	2.811	3.324
CHEMBL3326132	1.482	1.905	1.806
CHEMBL3326134	2.824	2.637	2.572
CHEMBL3326135	3.594	2.419	3.130
CHEMBL3326137	2.748	2.768	2.373
CHEMBL3326138	3.177	2.711	2.740
CHEMBL3326139	4.025	3.822	3.793
CHEMBL3326140	4.258	3.817	3.960
CHEMBL3326145	3.751	4.208	4.505
CHEMBL3326146	2.871	2.925	3.165
CHEMBL3326149	2.750	2.745	2.720
CHEMBL3326150	4.096	3.539	2.820
CHEMBL3326151	3.131	3.109	3.059
CHEMBL3393443	1.445	1.713	1.255
CHEMBL3393445	1.721	1.865	1.623
CHEMBL3393447	1.636	1.380	1.568
CHEMBL3393448	2.125	1.691	1.301
CHEMBL3393450	2.141	1.826	2.310
CHEMBL3393451	1.436	1.640	2.045
CHEMBL3393453	1.791	1.674	2.143

Table S2. NMDA data set. Predicted and experimental $LogIC_{50}$ values for ANN and BMLR model

ChEMBL ID	ANN-Pred_LogIC50[nM]	BMLR-Pred_LogIC50[nM]	Exp_LogIC50[nM]
CHEMBL12242	-0.039	0.281	0.079
CHEMBL12256	1.264		0.869
CHEMBL12301	1.034		2.079
CHEMBL12414	0.837	1.203	1.431
CHEMBL12513	0.655	0.315	-0.097
CHEMBL12546	2.250	1.809	2.114
CHEMBL126228	1.220	1.982	1.491
CHEMBL12706	3.073	2.747	2.398
CHEMBL12728	2.447		3.041
CHEMBL12733	1.194		2.204
CHEMBL12804	0.679		0.000

Table S2. *Cont.*

CHEMBL141985	3.088	3.364	3.204
CHEMBL145074	3.562	3.401	3.498
CHEMBL145362	3.172	3.467	3.223
CHEMBL145406	2.823	3.377	2.893
CHEMBL145728	2.915	3.235	2.836
CHEMBL146168	3.629	3.309	2.966
CHEMBL173031	2.736		2.029
CHEMBL17350	0.965		0.845
CHEMBL182066	1.430		0.778
CHEMBL182369	1.211	1.923	2.143
CHEMBL182665	1.573	1.245	0.903
CHEMBL182698	1.278	1.300	0.903
CHEMBL182951	1.281	1.384	1.230
CHEMBL183142	0.887	1.014	1.415
CHEMBL183457	0.967	1.298	1.230
CHEMBL183825	0.759	1.314	1.230
CHEMBL185812	1.192	1.331	1.732
CHEMBL21641	4.527	2.951	3.255
CHEMBL219631	1.014	1.085	0.602
CHEMBL22304	2.242	2.039	2.342
CHEMBL22720	3.557	3.163	3.519
CHEMBL268714	2.340		2.568
CHEMBL268920	1.107	1.522	0.987
CHEMBL269683	0.289	0.221	0.230
CHEMBL273636	1.015	0.632	0.903
CHEMBL273662	0.468	-0.076	0.176
CHEMBL273686	1.035	1.616	1.491
CHEMBL273889	1.869		2.934
CHEMBL274422	0.333	0.420	0.176
CHEMBL275906	0.263	0.897	0.908
CHEMBL275966	-0.018	0.913	1.230
CHEMBL276670	1.067	1.345	1.580
CHEMBL282003	2.961	2.554	2.447
CHEMBL282672	3.974	3.790	3.991
CHEMBL284028	3.350	2.475	2.114
CHEMBL288384	3.283	2.585	2.892
CHEMBL288839	2.254		1.863
CHEMBL288855	1.663		0.845
CHEMBL289167	3.643		3.633
CHEMBL289779	3.543	3.209	2.863
CHEMBL289832	2.722		0.771
CHEMBL290747	1.749	3.705	3.447
CHEMBL291148	2.719	2.214	1.978

Table S2. *Cont.*

CHEMBL291149	1.931	1.700	1.914
CHEMBL291161	2.371	2.362	2.342
CHEMBL291169	2.200		2.959
CHEMBL291170	2.949		3.519
CHEMBL295084	3.852	3.912	3.519
CHEMBL295154	3.188		1.724
CHEMBL295178	3.976	3.032	3.176
CHEMBL295995	2.406	2.126	2.176
CHEMBL296068	2.779	3.333	3.322
CHEMBL296521	2.420		3.491
CHEMBL297310	2.538	3.624	3.462
CHEMBL297881	2.319		0.672
CHEMBL298284	3.258		3.968
CHEMBL302783	2.324	2.192	2.664
CHEMBL305195	0.848		0.778
CHEMBL317229	1.187		0.602
CHEMBL357426	3.090	3.087	2.806
CHEMBL360373	1.505		1.204
CHEMBL360463	2.045	1.826	2.117
CHEMBL363010	0.795	1.327	0.602
CHEMBL363722	1.323	1.040	0.699
CHEMBL367816	1.510	1.025	1.602
CHEMBL39664	1.937	1.666	1.732
CHEMBL40623	2.107		1.079
CHEMBL40649	3.203	2.454	3.279
CHEMBL40652	2.165		1.462
CHEMBL40708	2.298	2.525	2.230
CHEMBL40745	2.448	1.978	2.114
CHEMBL40755	4.488	4.033	4.301
CHEMBL40976	1.804		1.362
CHEMBL41180	3.587	3.405	3.477
CHEMBL41291	2.277		2.041
CHEMBL41295	2.307	2.729	2.204
CHEMBL41340	2.819	2.820	2.462
CHEMBL41341	2.409	2.225	2.602
CHEMBL41399	2.092		2.806
CHEMBL41460	2.460		1.462
CHEMBL41501	4.261	3.967	3.732
CHEMBL41691	2.638	1.858	1.653
CHEMBL417710	3.963	3.407	4.114
CHEMBL41791	4.065		3.991
CHEMBL41939	4.131		4.279
CHEMBL42057	3.208		3.643

Table S2. *Cont.*

CHEMBL42071	3.477	3.625	3.146
CHEMBL42146	2.316		1.568
CHEMBL42176	1.890	1.720	2.342
CHEMBL42248	1.933	1.923	1.806
CHEMBL42307	1.480		3.602
CHEMBL42329	3.256	2.505	2.799
CHEMBL42350	3.301		3.176
CHEMBL42367	3.416		4.230
CHEMBL42426	2.201		0.940
CHEMBL42430	3.645	3.795	4.230
CHEMBL42466	2.416	2.175	2.176
CHEMBL42482	4.471	2.737	3.146
CHEMBL42694	3.345	3.435	3.255
CHEMBL42805	2.865	3.167	3.869
CHEMBL42915	1.840		1.519
CHEMBL43122	2.321		3.633
CHEMBL43318	3.086		3.531
CHEMBL43800	4.494		4.380
CHEMBL43846	2.138		1.477
CHEMBL43848	2.638		4.041
CHEMBL43869	4.570	3.936	4.204
CHEMBL44018	2.591		3.556
CHEMBL442545	3.491		4.176
CHEMBL44260	3.630	3.425	3.255
CHEMBL536106	0.519		1.903
CHEMBL536107	1.362		0.301
CHEMBL537478	-0.339		0.176
CHEMBL541604	3.504	3.374	3.023
CHEMBL543252	2.358	1.685	1.813
CHEMBL543489	0.964		1.778
CHEMBL543722	2.922	1.745	1.602
CHEMBL544390	4.180	3.632	4.001
CHEMBL552664	0.502	0.712	0.477
CHEMBL553334	2.779	2.698	3.000
CHEMBL557993	1.716		0.613
CHEMBL84612	2.353	2.155	2.248

Table S3. TrkA data set. Predicted and experimental $LogIC_{50}$ values for ANN and BMLR model

ChEMBL ID	ANN-Pred_LogIC50[nM]	BMLR-Pred_LogIC50[nM]	Exp_LogIC50[nM]
CHEMBL3671129	0.067	0.044	-0.155
CHEMBL3671130	-0.075	0.271	-0.155
CHEMBL3671131	-0.197	0.194	0.362
CHEMBL3671132	0.060	0.081	-0.022
CHEMBL3671133	0.160	-0.007	-0.022
CHEMBL3671134	0.159	0.299	0.190
CHEMBL3671135	0.134	0.001	-0.347
CHEMBL3671137	0.342	0.624	0.538
CHEMBL3671138	-0.087	0.092	0.021
CHEMBL3671141	-0.193	0.018	-0.301
CHEMBL3671142	0.079	0.063	-0.260
CHEMBL3671144	-0.030	0.162	-0.046
CHEMBL3671145	0.040	0.308	0.332
CHEMBL3671146	1.410	1.593	1.583
CHEMBL3671147	1.729	1.860	1.871
CHEMBL3671148	-0.047	0.044	-0.222
CHEMBL3671149	0.541	0.479	0.470
CHEMBL3671150	0.208	0.343	0.322
CHEMBL3671152	0.639	0.593	0.556
CHEMBL3671154	-0.090	0.301	0.114
CHEMBL3671156	0.317	0.562	0.447
CHEMBL3671157	0.101	0.172	0.114
CHEMBL3675908	0.022	0.173	0.290
CHEMBL3675914	0.061	0.643	0.322
CHEMBL3675915	-0.134	0.737	0.217
CHEMBL3675918	-0.015	0.138	-0.097
CHEMBL3675922	0.344	0.121	0.176
CHEMBL3675923	-0.023	0.287	0.146
CHEMBL3675924	0.248	0.270	0.519
CHEMBL3675925	0.015	-0.067	0.176
CHEMBL3675928	0.177	0.011	0.072
CHEMBL3675929	0.863	0.921	0.869
CHEMBL3675930	0.782	1.039	1.017
CHEMBL3675931	0.036	0.626	0.352
CHEMBL3675932	0.109	0.964	0.380
CHEMBL3675933	0.238	0.618	0.594
CHEMBL3675934	-0.001	0.664	0.973
CHEMBL3675936	0.178	0.481	0.352
CHEMBL3675937	0.014	0.251	0.290
CHEMBL3675938	0.050	0.236	0.403
CHEMBL3675939	-0.088	0.104	0.658
CHEMBL3675940	-0.093	0.006	0.204

Table S3. *Cont.*

CHEMBL3675941	-0.166	0.045	-0.222
CHEMBL3675942	0.056	0.037	-0.244
CHEMBL3675943	0.312	0.254	0.708
CHEMBL3675944	0.903	2.090	1.780
CHEMBL3675945	0.490	1.131	1.379
CHEMBL3675946	0.627	1.045	0.937
CHEMBL3675949	0.522	0.942	1.100
CHEMBL3675950	-0.100	0.663	0.842
CHEMBL3675952	0.245	0.647	0.730
CHEMBL3675959	0.113	0.979	1.323
CHEMBL3675960	0.338	0.674	0.972
CHEMBL3675962	0.431	0.814	1.207
CHEMBL3675964	0.036	0.707	0.556
CHEMBL3675967	2.404	2.730	3.006
CHEMBL3675968	0.771	1.030	0.708
CHEMBL3675969	2.797	2.864	2.755
CHEMBL3675970	0.907	0.768	0.732
CHEMBL3675971	2.431	2.928	2.535
CHEMBL3675972	0.678	0.693	0.792
CHEMBL3675973	0.684	0.884	0.954
CHEMBL3675974	0.902	1.039	0.845
CHEMBL3675975	0.895	1.040	1.068
CHEMBL3675977	1.211	1.601	1.943
CHEMBL3675978	0.984	1.651	1.920
CHEMBL3675979	1.158	0.876	1.405
CHEMBL3675980	0.942	1.135	0.886
CHEMBL3675981	1.011	0.905	1.215
CHEMBL3675982	2.839	3.009	3.076
CHEMBL3675984	1.058	1.242	1.571
CHEMBL3675985	1.043	1.354	1.577
CHEMBL3675986	1.295	1.115	1.490
CHEMBL3675987	0.479	0.835	0.505
CHEMBL3675988	1.138	1.067	1.476
CHEMBL3675989	1.262	1.079	1.185
CHEMBL3675990	1.104	1.016	1.418
CHEMBL3675991	1.059	1.251	1.653
CHEMBL3675992	0.809	1.391	1.352
CHEMBL3675994	1.216	1.635	1.358
CHEMBL3675996	0.664	1.146	1.521
CHEMBL3675997	0.608	0.760	0.806
CHEMBL3675999	1.215	1.241	1.107
CHEMBL3676000	2.496	2.420	2.339
CHEMBL3676001	2.897	2.558	2.672

Table S3. *Cont.*

CHEMBL3676003	0.698	1.181	0.903
CHEMBL3676005	0.719	0.979	0.699
CHEMBL3676006	1.121	1.377	1.647
CHEMBL3676007	1.157	1.480	1.204
CHEMBL3676008	0.951	0.937	0.869
CHEMBL3676010	0.646	1.553	1.420
CHEMBL3676011	2.712	2.672	2.900
CHEMBL3676012	0.815	1.174	1.542
CHEMBL3676015	0.911	1.039	0.973
CHEMBL3676016	1.221	1.119	1.104
CHEMBL3676017	0.847	0.888	0.903
CHEMBL3676020	0.638	0.953	0.785
CHEMBL3676021	0.685	0.883	0.756
CHEMBL3676023	1.165	0.965	1.258
CHEMBL3676024	0.701	0.824	0.322
CHEMBL3676029	0.805	1.307	0.996
CHEMBL3676030	0.484	0.889	0.633
CHEMBL3676034	0.015	0.005	0.041
CHEMBL3676035	0.077	0.286	0.380
CHEMBL3676036	0.031	0.175	0.342
CHEMBL3676037	0.057	0.064	0.079
CHEMBL3676038	0.037	0.017	-0.046
CHEMBL3676040	0.118	0.307	0.672
CHEMBL3676041	-0.005	0.154	0.398
CHEMBL3676042	0.066	0.106	0.176
CHEMBL3676045	0.163	0.113	0.255
CHEMBL3676046	0.000	0.232	0.398
CHEMBL3676049	1.205	1.166	1.233
CHEMBL3676050	0.426	0.846	1.053
CHEMBL3676051	0.666	0.960	0.839
CHEMBL3676052	0.700	0.861	0.869
CHEMBL3676053	0.843	0.981	0.934
CHEMBL3676060	2.637	2.742	2.641
CHEMBL3676061	1.143	1.080	1.193
CHEMBL3676062	0.720	0.987	0.886
CHEMBL3676063	0.967	0.967	0.833
CHEMBL3676066	0.742	1.000	0.556
CHEMBL3676067	0.688	0.860	1.104
CHEMBL3676069	0.767	0.714	1.286
CHEMBL3676071	0.190	0.799	1.155
CHEMBL3676072	0.304	0.720	0.591
CHEMBL3676075	0.613	0.892	1.233
CHEMBL3676076	0.319	0.718	0.519

Table S3. *Cont.*

CHEMBL3676077	0.209	0.791	0.623
CHEMBL3676078	0.778	1.336	1.580
CHEMBL3676079	1.105	1.265	0.934
CHEMBL3676080	1.130	1.226	1.185
CHEMBL3676081	0.030	0.816	0.491
CHEMBL3676082	0.362	0.673	0.763
CHEMBL3676086	1.481	1.760	1.693
CHEMBL3676088	0.771	0.569	1.021
CHEMBL3676089	0.978	0.601	1.090
CHEMBL3676090	0.823	0.684	1.114
CHEMBL3676091	0.911	0.944	1.201
CHEMBL3676092	0.885	0.586	0.477
CHEMBL3676093	0.823	1.198	0.940
CHEMBL3676094	1.010	0.762	0.653
CHEMBL3676096	0.709	1.088	1.029
CHEMBL3676097	0.568	0.708	0.447
CHEMBL3676098	0.586	0.724	0.342
CHEMBL3676099	0.690	0.670	0.322
CHEMBL3676100	0.644	0.572	0.279
CHEMBL3676102	1.017	1.258	1.053
CHEMBL3676103	1.056	1.137	1.338
CHEMBL3676104	0.797	1.137	0.949
CHEMBL3676105	1.116	1.177	0.954

Table S4. Binding free energies (in kcal/mol) of the NMDA–ligand complexes calculated using the MM/GBSA method.

**Energy term**	**NMDA**			
	GNE-5729	**1N**	**2N**	**3N**
ΔE_{H-bnd}	-0.33	-0.80	-0.28	-0.25
$\Delta E_{covalent-bnd}$	1.52	0.94	-1.33	1.76
ΔE_{el}	-21.94	-12.16	-17.25	-7.22
ΔE_{vdW}	-65.43	-52.99	-52.62	-49.51
$\Delta E_{\pi-\pi}$	-1.75	-5.02	-0.19	-0.74
ΔG_{pol}	23.21	25.77	17.88	19.06
ΔG_{np}	-52.52	-34.24	-33.28	-29.50
ΔG_{bind}	-117.25	-78.50	-87.07	-66.39
Ligand efficiency	4.34	3.14	3.63	2.89

Table S5. Binding free energies (in kcal/mol) of the LRRK2–ligand complexes calculated using the MM/GBSA method.

Energy term	LRRK2			
	PF-06447475	**1L**	**2L**	**3L**
ΔE_{H-bnd}	-0.97	-0.38	-0.78	-0.23
$\Delta E_{covalent-bnd}$	2.11	2.17	1.95	1.34
ΔE_{el}	-14.22	-12.78	-5.62	-5.13
ΔE_{vdW}	-43.48	-54.23	-40.02	-43.08
$\Delta E_{\pi-\pi}$	-1.69	-5.35	-0.22	-0.01
ΔG_{pol}	14.74	14.51	13.65	13.73
ΔG_{np}	-28.91	-49.33	-32.37	-35.74
ΔG_{bind}	-72.41	-100.04	-63.40	-69.12
Ligand efficiency	3.15	4.00	2.76	3.01

Table S6. Binding free energies (in kcal/mol) of the TrkA–ligand complexes calculated using the MM/GBSA method.

Energy term	TrkA			
	**AZ-23**	**1T**	**2T**	**3T**
ΔE_{H-bnd}	-0.50	-0.36	0	-0.26
$\Delta E_{covalent-bnd}$	4.51	2.59	1.34	0.88
ΔE_{el}	-10.80	-4.65	-11.97	-2.21
ΔE_{vdW}	-45.38	-26.88	-26.89	-46.04
$\Delta E_{\pi-\pi}$	-0.16	0	-0.09	-0.13
ΔG_{pol}	16.86	16.58	23.39	17.15
ΔG_{np}	-34.96	-23.42	-28.77	-39.19
ΔG_{bind}	-70.44	-36.13	-43.01	-69.81
Ligand efficiency	2.61	1.39	2.69	2.79

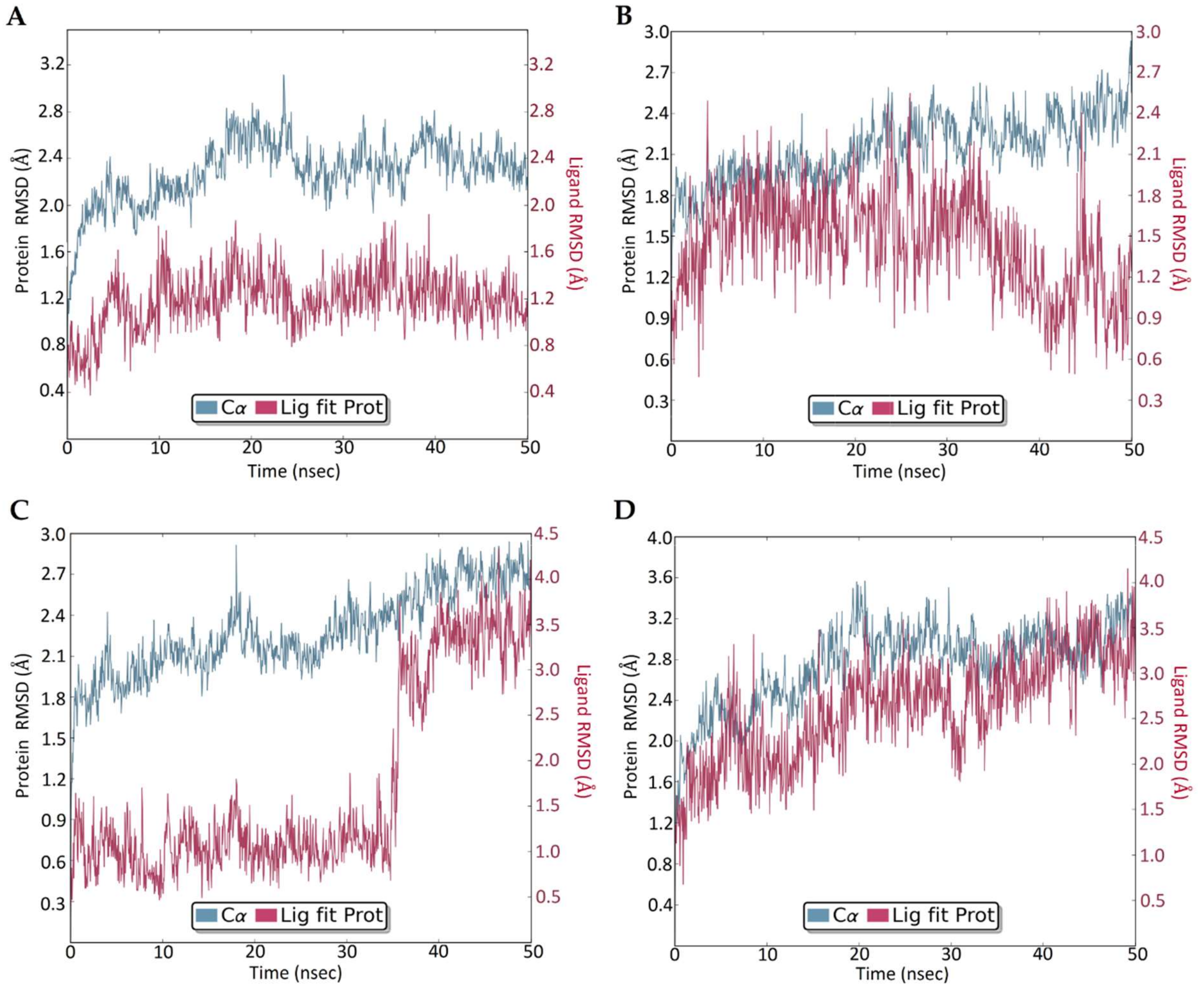

Figure S1. RMSD of the atomic positions for the compounds GNE-5729 (A), **1N (B), 2N (C)** and **3N (D)** (in red) and the receptor NMDA (in blue) of the 50 ns molecular dynamics simulations using Desmond code.

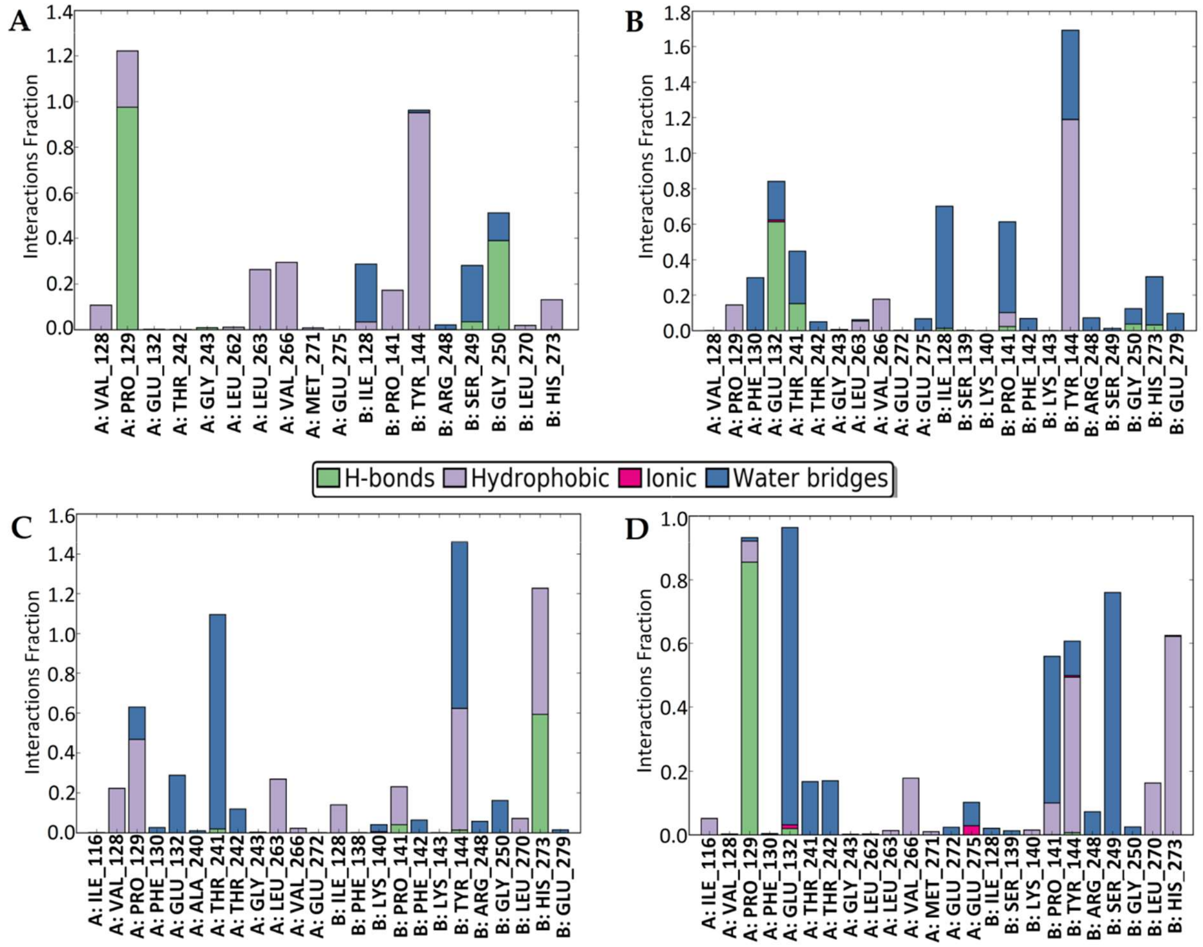

Figure S2. Molecular dynamics calculated contacts between compounds GNE-5729 (A), **1N (B), 2N (C)** and **3N (D)** and NMDA.

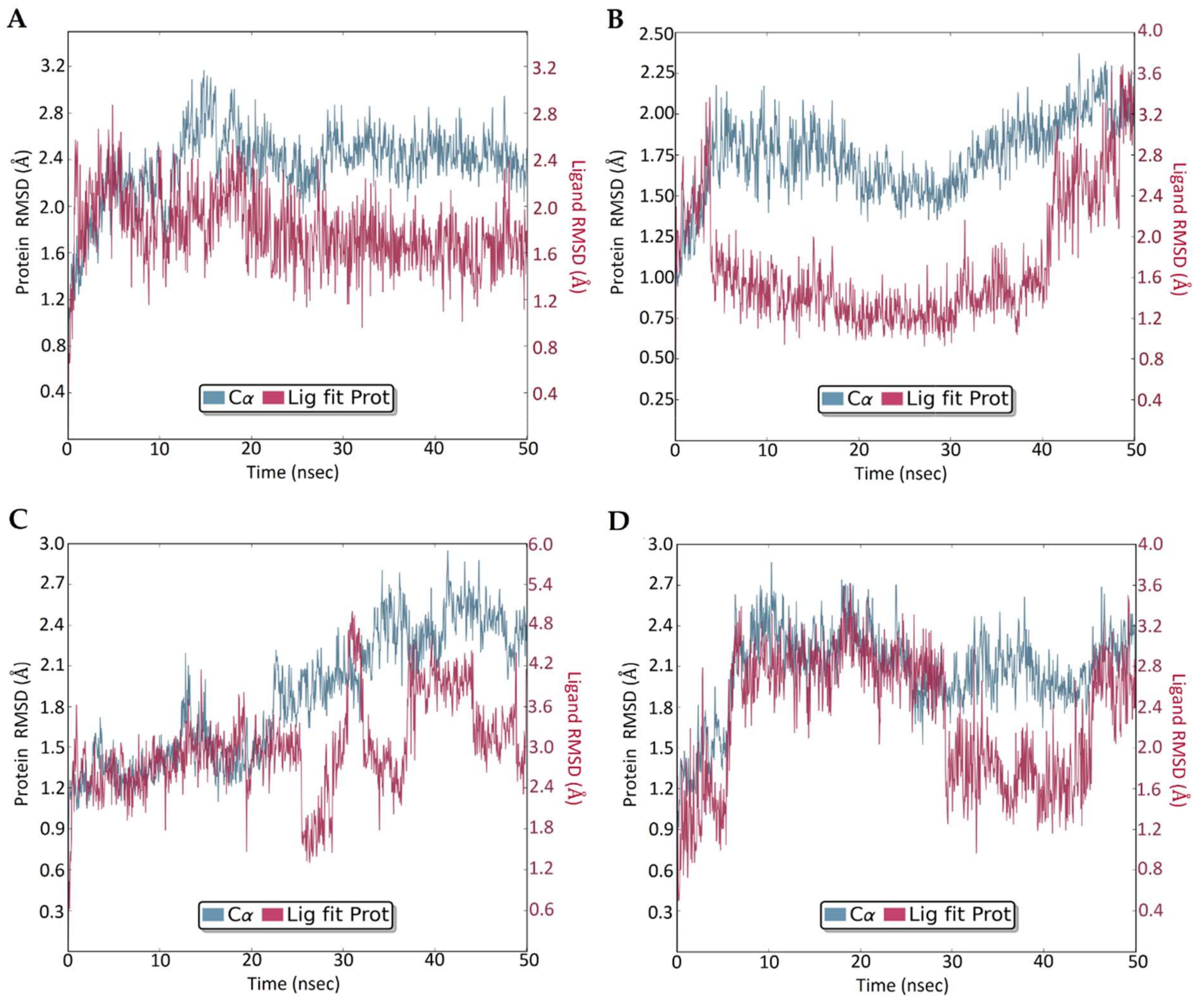

Figure S3. RMSD of the atomic positions for the compounds PF-06447475 **(A), 1L (B), 2L (C)** and **3L (D)** (in red) and the receptor LRRK2 (in blue) of the 50 ns molecular dynamics simulations using Desmond code.

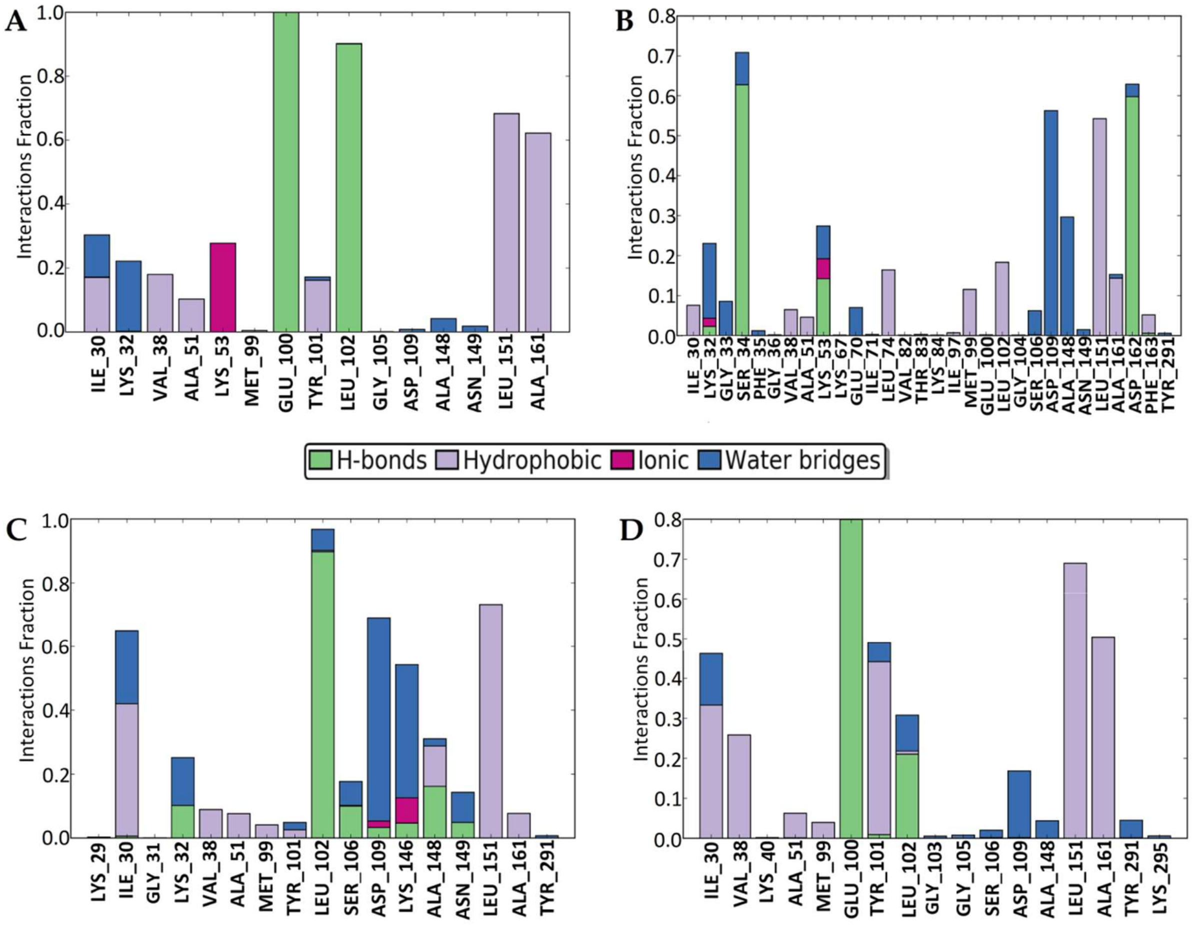

Figure S4. Molecular dynamics calculated contacts between compounds PF-06447475 (A), **1L (B), 2L (C), 3L (D)** and LRRK2.

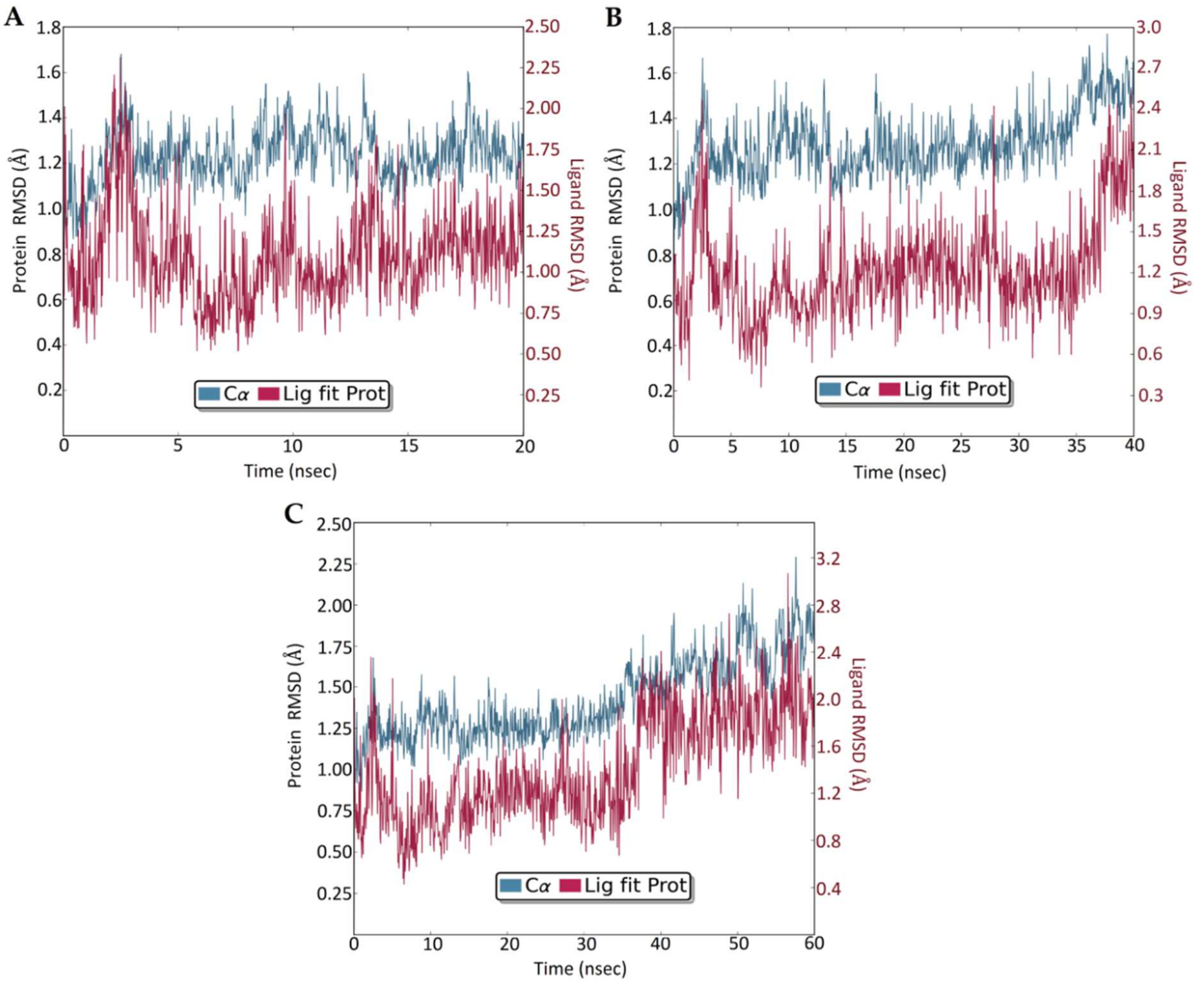

Figure S5. RMSD of the atomic positions for the compounds **1L** (in red) and the receptor LRRK2 (in blue) of the 20 ns **(A),** 40 ns **(B)** and 60 ns **(C)** molecular dynamics simulations using Desmond code.

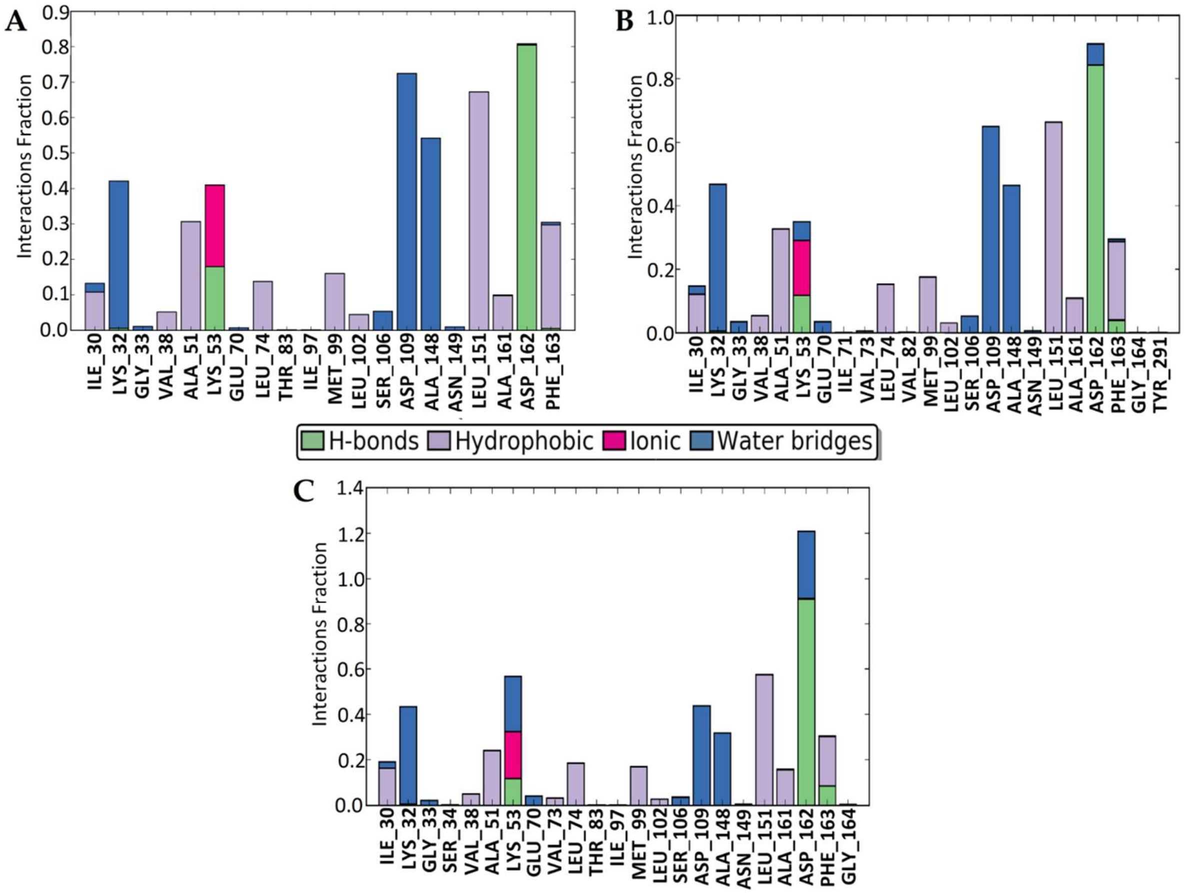

Figure S6. Molecular dynamics calculated contacts between compound **<u>1L</u>** and LRRK2 **(A)** – 20 ns, **(B)** – 40 ns, **(C)** – 60 ns.

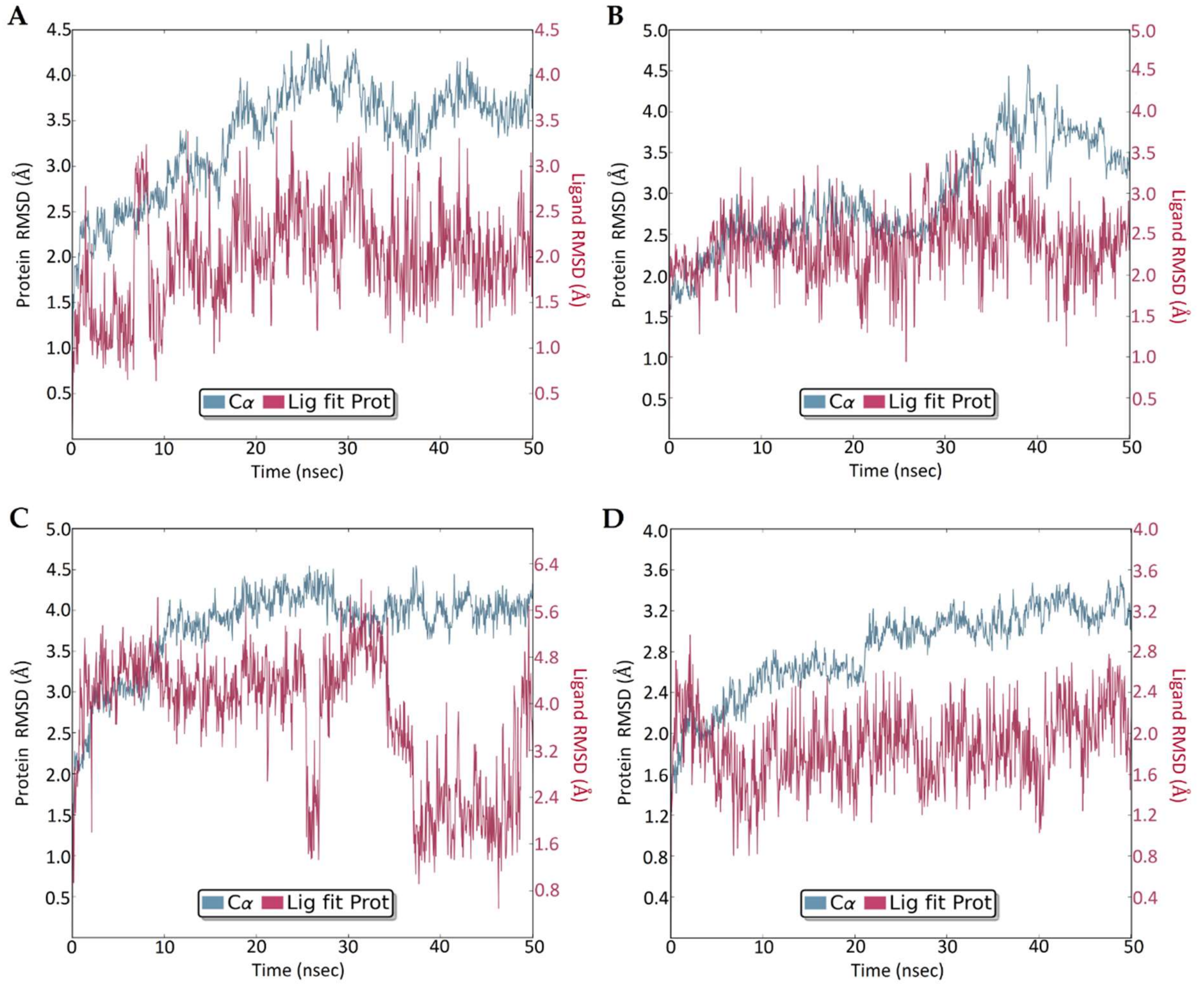

Figure S7. RMSD of the atomic positions for the compounds **AZ-23 (A)**, **1T (B), 2T (C)** and **3T (D)** (in red) and the receptor TrkA (in blue) of the 50 ns molecular dynamics simulations using Desmond code.

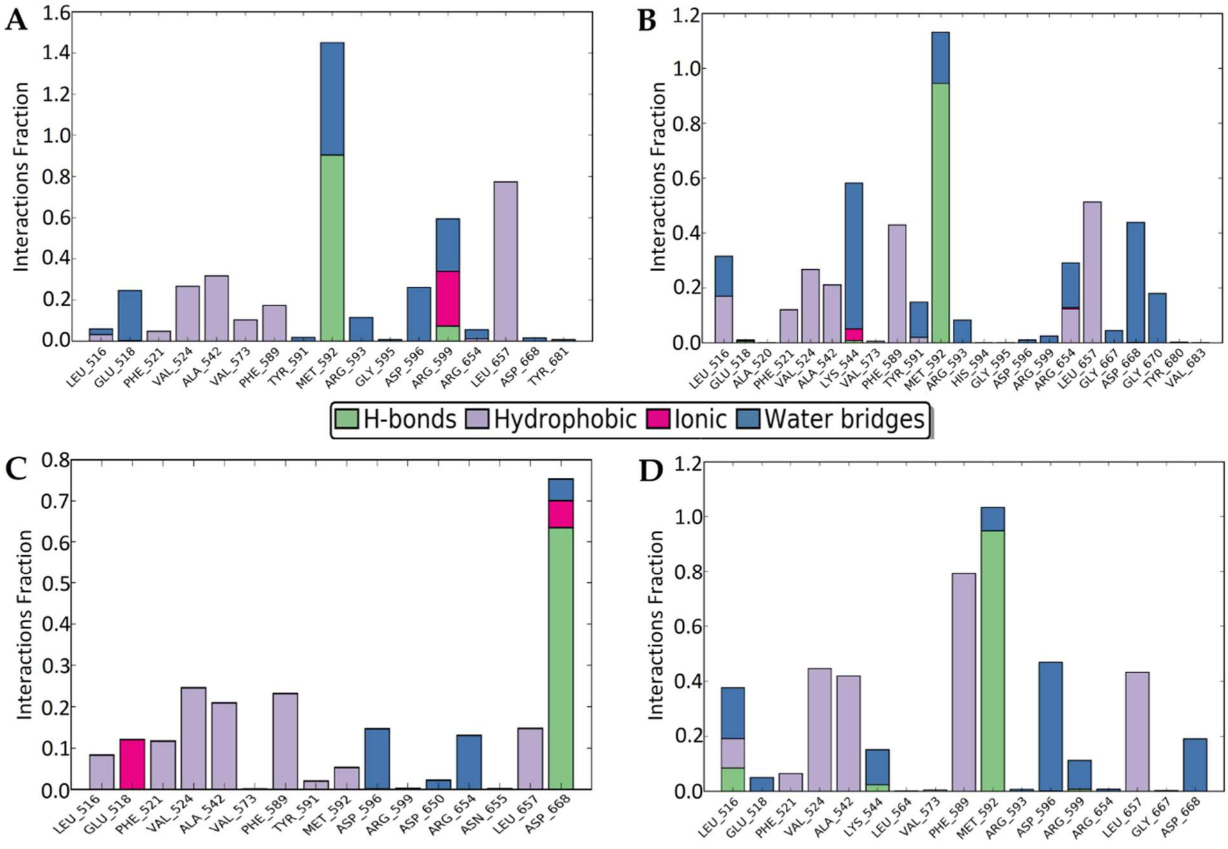

Figure S8. Molecular dynamics calculated contacts between compounds **AZ-23 (A)**, **1T (B), 2T (C)**, **3T (D)** and TrkA.
